# Therapeutic applications and potential mechanisms of acupuncture in migraine: A literature review and perspectives

**DOI:** 10.3389/fnins.2022.1022455

**Published:** 2022-10-20

**Authors:** Ying Chen, Yuhan Liu, Yine Song, Shaoru Zhao, Bin Li, Jingqing Sun, Lu Liu

**Affiliations:** Department of Acupuncture and Moxibustion, Beijing Hospital of Traditional Chinese Medicine, Beijing Key Laboratory of Acupuncture Neuromodulation, Capital Medical University, Beijing, China

**Keywords:** acupuncture, migraine, neural mechanism, neuroinflammation, neuronal sensitization, neuroimaging, review

## Abstract

Acupuncture is commonly used as a treatment for migraines. Animal studies have suggested that acupuncture can decrease neuropeptides, immune cells, and proinflammatory and excitatory neurotransmitters, which are associated with the pathogenesis of neuroinflammation. In addition, acupuncture participates in the development of peripheral and central sensitization through modulation of the release of neuronal-sensitization-related mediators (brain-derived neurotrophic factor, glutamate), endocannabinoid system, and serotonin system activation. Clinical studies have demonstrated that acupuncture may be a beneficial migraine treatment, particularly in decreasing pain intensity, duration, emotional comorbidity, and days of acute medication intake. However, specific clinical effectiveness has not been substantiated, and the mechanisms underlying its efficacy remain obscure. With the development of biomedical and neuroimaging techniques, the neural mechanism of acupuncture in migraine has gained increasing attention. Neuroimaging studies have indicated that acupuncture may alter the abnormal functional activity and connectivity of the descending pain modulatory system, default mode network, thalamus, frontal-parietal network, occipital-temporal network, and cerebellum. Acupuncture may reduce neuroinflammation, regulate peripheral and central sensitization, and normalize abnormal brain activity, thereby preventing pain signal transmission. To summarize the effects and neural mechanisms of acupuncture in migraine, we performed a systematic review of literature about migraine and acupuncture. We summarized the characteristics of current clinical studies, including the types of participants, study designs, and clinical outcomes. The published findings from basic neuroimaging studies support the hypothesis that acupuncture alters abnormal neuroplasticity and brain activity. The benefits of acupuncture require further investigation through basic and clinical studies.

## Introduction

Migraine is an episodic, recurrent dysfunction of brain excitability with the hallmark of moderate-to-severe unilateral throbbing and pulsating headaches (Noseda and Burstein, 2013). The latest Global Burden of Disease Study showed that 1.25 billion individuals experienced migraine attacks in a year. Overall, migraine is the fifth most prevalent and seventh most incapacitating condition worldwide. Various pharmacological and non-pharmacological remedies are typically used to alleviate severe pain or avoid migraine attacks. Drugs such as triptans, propranolol, ergotamine preparations, flunarizine, and valproic acid appear beneficial for treating migraine. However, they all have side effects with long-term use (Silberstein et al., 2012).

Acupuncture, a widely used non-pharmacological therapy, offers the benefit of therapeutic results and few adverse effects in the prevention and treatment of migraines (Xu et al., 2018; Chen et al., 2020). The latest Cochrane review of acupuncture pointed out that acupuncture as migraine prophylaxis may be at least as effective as medication therapy in preventing migraine and is more beneficial than sham acupuncture (Linde et al., 2016). Nevertheless, its specific clinical effectiveness remains controversial, and the mechanism of acupuncture’s effectiveness remains obscure.

In recent years, the number of clinical trials of acupuncture therapy for migraines has increased, as has research into the neural mechanism. Clinical, basic, and neuroimaging studies vary considerably in type, yet few papers have thoroughly reviewed these studies. The parameters of the study design and the corresponding findings of the brain mechanisms have not been thoroughly studied or presented in the available reviews.

The pathophysiology of migraine has yet to be completely expounded, but the importance of neuroinflammation as an initiator and driver of migraine attacks has been advocated for decades (Moskowitz, 1993). The headache is caused by activation of the trigeminovascular system (Ashina et al., 2019), which is followed by the release of neuropeptides into the perivascular space (Pietrobon and Moskowitz, 2013), resulting in an inflammatory cascade reaction (Levy, 2009). The persistent neuroinflammation could prompt neuronal sensitization, finally leading to persistent impairment of brain functioning (Kursun et al., 2021). Emerging data from animal models and human imaging studies have proven the neuroinflammation pathway in the pathophysiology of migraines (Albrecht et al., 2019; Hadjikhani et al., 2020). In the basic studies we included, the findings mainly pertained to the regulation of neuroinflammation and neuronal sensitization by acupuncture. It may be a potential mechanism for how acupuncture effects migraines.

Additionally, as a disabling neurological condition, abnormality in the structure and functioning of the brain have also been found to be contributors to migraines (Hougaard et al., 2014; Schwedt et al., 2015a). The advancements in neuroimaging techniques have provided improved insights into the neural mechanisms underlying not only the occurrence and development of migraines, but also the impact of acupuncture on patients with migraines.

Therefore, in this review, we summarize the evidence from clinical, animal, and neuroimaging trials to explore the characteristics of current studies and discuss the effects of acupuncture in preventing neuroinflammation and neuronal sensitization, abnormal brain structure, and functioning in migraines, which has the potential to reveal the neural mechanisms underlying the effects of acupuncture in the prevention and treatment of migraines.

## Methods

### Search strategies

We searched for studies published between September, 1965 and 2022 in the PubMed, Science Direct, and Web of Science databases The last search was conducted on March 30, 2022. The keywords used for search included “Acupuncture” and “Migraine.” This search strategy yielded a total of 1067 articles, 645 of which were from PubMed, 119 from Science Direct and 303 from Web of Science.

### Inclusion criteria

We included controlled clinical trials conducted on people with migraines, as well as basic research on migraine models published in English.

### Exclusion criteria

We excluded articles that were published in other languages, or irrelevant to acupuncture and migraines. Article types other than controlled clinical trials including comments, review articles, meta-analyses, protocols and case reports were also excluded.

### Study selection

**FIGURE 1:**
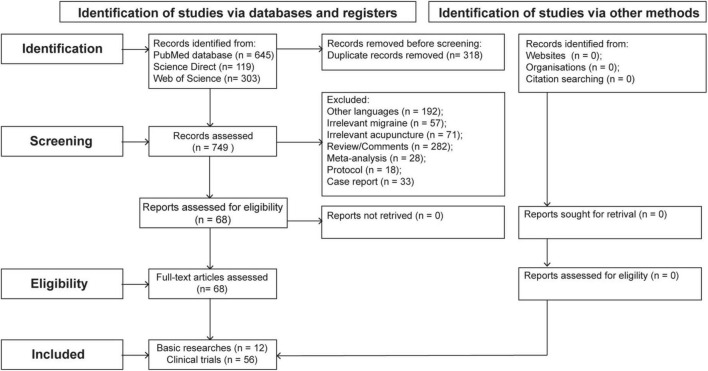
Flow diagram of the review processes.

Three hundred and eighteen articles were excluded because of duplication, the remaining 749 articles were analyzed according to their titles and abstracts and of these articles, 681 articles were further excluded as follows: 192 articles were excluded because of publicated in other languages, 57 articles were excluded because of irrelevance to migraine, 71 articles were excluded because of irrelevance to acupuncture, 282 articles were excluded because of they were comments or review articles, 28 articles were excluded because of they were meta-analyses, 18 articles were excluded because of they were protocols, 33 articles were excluded because of they were case reports. The full texts of the remaining 68 articles were obtained and analyzed in depth. Ultimately, this process identified 56 clinical (including 40 neuroimaging studies) and 12 basic trials. Figure 1 illustrates the flowchart of the search process.

### Data extraction

Two authors independently evaluated the titles and abstracts of the retrieved and full-text articles. The final articles’ data were validated and extracted according to the predefined criteria extraction table, enumerating the trial design, intervention, comparison, acupoints, acupuncture parameters and outcomes. Any disagreements were resolved through discussions between the authors.

## Clinical study status and efficacy of acupuncture in migraine patients

**FIGURE 2:**
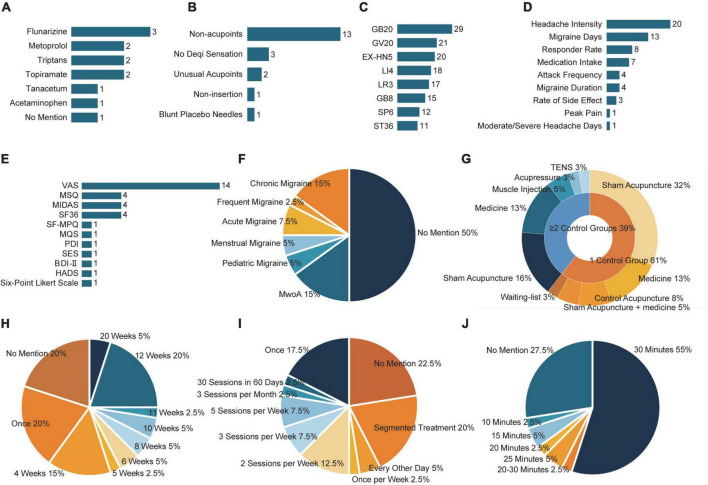
The study design of included clinical studies. **(A)** The number of detailed medication usage. **(B)** The number of detailed sham acupuncture techniques. **(C)** The frequency of selected acupoints. **(D)** The frequency of improved migraine-related outcomes after acupuncture. **(E)** The frequency of improved migraine-related scales after acupuncture. **(F)** The proportion of subtypes of migraine. **(G)** The proportion of control group types. **(H)** The proportion of treatment frequency. **(I)** The proportion of the total duration of acupuncture treatment. **(J)** The proportion of the retaining time. BDI, Beck Depression Inventory; EX-HN, Tojingbu Xue Points of Head and Neck; GB, gallbladder meridian; GV, governor vessel; HADS, Hospital Anxiety and Depression Scale; LI, large intestine meridian; LR, liver meridian; MIDAS, Migraine Disability Assessment scale; MQS, Medication Quantification Scale; MwoA, migraine without aura; PDI, Pain Disability Index; SES, Schmerzempfindungsskala (scale for assessing emotional aspects of pain); SF-MPQ, Short Form of McGill Pain Questionnaire; SF-36, Short Form-36; SP, spleen meridian; ST, stomach meridian; VAS, Visual Analog Scale.

Forty non-imaging-related clinical trials were included, totaling 5576 participants. The characteristics of the 40 included clinical studies are summarized in Supplementary Table 1. The study designs are compared in Figure 2.

### Characteristics of current clinical studies

#### Participants

Among these 40 trials, 22 did not describe the subtypes of migraine. Seven focused on migraine without aura (MwoA), six on chronic migraine (CM), two on pediatric migraine, two on pure menstrual and menstrually related migraine, and three on acute migraine attack (Figure 2F).

#### Control groups

Except for two before-after trials, 38 trials used a parallel-group design. Of these, 23 trials included two groups (acupuncture plus a control group), 14 included three groups, and one had four. Detailed control group information is presented in Figure 2G.

A total of 10 trials used medication alone as a control, and four used control acupuncture combined with medication. The most commonly used medicine was flunarizine (Figure 2A). Two trials used intramuscular injection (botulinum toxin A), one trial used psychotherapy (hypnotherapy), one trial used usual care, one used transcutaneous electrical nerve stimulation with laser therapy, and four trials used a wait list. The sham acupuncture technique was applied as a control in 20 trials. The most commonly used sham acupuncture technique was stimulating non-acupoints (Figure 2B).

#### Interventions

There were various types of acupuncture methods in the included studies: 35 trials were conducted with manual acupuncture (MA), one with electrical stimulation (sparse-dense wave at a frequency of 100 Hz), three with auricular acupuncture (two used semi-permanent needles, one used standard acupuncture needles), and one with acupoint injection (botulinum toxin A). The frequency and total number of acupuncture treatments varied among studies. In most studies, the treatment time ranged from 4 to 12 weeks (Figure 2H), 1–5 sessions per week (Figure 2I), except for seven studies with only one treatment. The needling details were also reported with sufficient information, including the retention time ranging from 10 to 30 min (Figure 2J), while the insertion depth ranged from 2 to 30 mm. The parameters above are not regulated in clinical treatment, and researchers have carried out the treatment according to their experience.

#### Acupoints

In most trials, acupuncture points were chosen based on the diagnosis and identification of symptom patterns were combined with a preset list of acupuncture points as part of a semi-standardized therapy protocol. Four trials administered individualized treatment to patients based on their diagnosis. A combination of body and scalp acupoints were most often employed in patients with migraine, with scalp acupoints situated on the side and top of the head. The most selected acupoints on the head were GB20 and GV20, and EX-HN5. The selection of body points varied and included acupoints on the neck, arms, legs, and abdomen. LI4 was the most selected acupoint, followed by LR3, SP6, and ST36 (Figure 2C).

#### Outcomes

The following clinical outcome measures showed substantial improvement: headache intensity, headache frequency, migraine attack duration, migraine or headache days, and responder rates. Concerning the scale results, studies mainly showed pain intensity using the Visual Analog Scale (VAS), combined with the McGill Pain Questionnaire Short Form (SF-MPQ), and a six-point Likert scale. Furthermore, the global improvement in migraine impact was measured by considering the quality of life, mental health, and disability. Studies have evaluated the effect of acupuncture on quality of life using the Migraine-Specific Quality of Life Questionnaire (MSQ) and Short Form-36 Health Survey Scale (SF-36). The Beck Depression Inventory, Hospital Anxiety and Depression Scale, and Self-Esteem Scale were used to assess psychiatric comorbidities in migraine patients. The disability score was evaluated using the Pain Disability Index and Migraine Disability Assessment Scale (Figures 2D,E).

### Clinical efficacy of acupuncture treatment on migraine

#### Headache-related indicators

A total of 33 studies reported positive clinical results for migraine-related symptoms. In these studies, acupuncture showed persistent, exceptional, and clinically relevant benefits for migraine in terms of lessening the severity of the headache (e.g., VAS, SF-MPQ, and Six-Point Likert Scale), migraine attack duration, headache frequency, the number of headache days, and the days of acute medication use. The long-term and instant effects of acupuncture have been demonstrated in randomized controlled trials. In light of their findings, the verum acupuncture group showed a significant decrease in migraine frequency, migraine days, and headache intensity (Wang et al., 2012) compared to placebo acupuncture, as well as a long-term improvement in the emotional domains of quality of life (Zhao L. et al., 2017). The systematic review by Linde et al. (2016), which described several new trials that demonstrated that acupuncture is superior to conventional pharmaceutical therapy in preventing migraines.

In our research, nine of ten trials with medication control showed acupuncture had more favorable effects when compared to medication. In comparison to migraine medications, acupuncture had a superior long-term impact. Compared with flunarizine, acupuncture has been suggested to be more effective in decreasing the frequency, severity, and duration of pain (Allais et al., 2002; Streng et al., 2006). According to two trials, acupuncture was significantly superior to topiramate in lowering the number of moderate/severe headache days and the days of acute medication intake (Yang et al., 2011, 2013). Streng et al.’s study indicated that acupuncture resulted in a greater decrease in the average pain scale and number of migraine attacks than metoprolol (Streng et al., 2006). In contrast to acute medications, the acupuncture-treated group showed a lower pain intensity than those treated with acetaminophen (Tastan et al., 2018), while Melchart et al. (2003) found that sumatriptan was more effective than acupuncture in relieving headaches in the first 2 h. Among the clinical trials we included, there are plenty of studies confirmed that acupuncture brings better benefits than prophylactic drugs, yet the effect on acute pain relief still need further exploration.

#### Relevant comorbidity and quality of life

Migraines are associated with a wide range of comorbidities and have a higher prevalence of many medical disorders. Neurological comorbidities include epilepsy, restless legs syndrome, sleep disorders, and ischemic stroke. Asthma, allergic rhinitis, vascular issues, and non-headache pain syndromes (e.g., fibromyalgia and temporomandibular joint disease) are examples of medical comorbidities (Buse et al., 2010, 2013; Lipton et al., 2016; Minen et al., 2016).

Psychiatric comorbidities include depression, anxiety, and suicidality. About 40% of migraineurs also mention depression (Lipton et al., 2000), and approximately 50% have an overall incidence of anxiety disorders (Minen et al., 2016). Acupuncture has decreased anxiety and depression and increased self-esteem in migraineurs (Wang et al., 2021).

Migraines, along with the comorbidities, can affect the quality of life and improve the disability rate of migraineurs. Studies have reported improvement in quality of life through the falling score of the MSQ and the induction of SF-36 (Cayir et al., 2014; Xu et al., 2020), and all of the trials that evaluated disability conditions showed changes in the relevant disability scores (Streng et al., 2006; Facco et al., 2008). The relationship between migraine and comorbidity, decreased quality of life, and disability is bidirectional (Breslau et al., 1994; Lipton et al., 1995). Therefore, it is necessary to control comorbidities and improve the quality of life in patients with migraine, which needs to be discussed in further acupuncture trials.

### Potential mechanism underlying acupoints analgesia

In traditional Chinese medicine theory, acupoints near an area of concern are considered to treat that concern. In clinical applications, scalp acupoints are commonly used for treating migraine (Wang et al., 2019). Scalp acupoints have been speculated to directly activate and modify the corresponding functional regions of the cerebral cortex and their blood flow (Wang J. et al., 2017). Most importantly, scalp acupoints may play an role in migraine treatment through trigeminal relevant connection. Wang et al. (2020) inferred the convergence and interaction of inputs from face tissues and the dura mater by the recordance of wide dynamic range (WDR) neurons in the spinal trigeminal nucleus caudalis (TNC) and the detection of bifurcated axons of the trigeminal ganglion. Head and face acupoints are directly connected to intracranial structures through the presynaptic dorsal root reflex, postsynaptic neurogenic responses, and convergence. Moreover, Wang et al. explored the contact between scalp acupoints and the meninges through observing the neurogenic plasma extravasation on a rat’s facial skin and dura mater after stimulation of the trigeminal nerves. The findings suggested that axon branches of the primary neurons in the TG may innervate scalp acupoints and the leptomeninges, modulating nutrients and active compounds in brain tissue (Moskowitz et al., 1988; Wang S. et al., 2017). Therefore, scalp acupuncture may have the impact of shortening the distance of the axon reflexes from the primary neurons in the TG.

Acupuncture has analgesic effects locally (scalp acupoints) and remotely (body acupoints), which may be mediated by different mechanisms. The selection of body points on the arms and legs is often used in treating migraine. The chemical mechanism of ST36 has been explored for its remote clinical effects. Acupuncture near ST36 can significantly increase the local concentration of extracellular adenosine, a neuromodulator with neuroprotective, anti-inflammatory, and analgesic properties (Goldman et al., 2010). Previous studies have demonstrated that locally produced adenosine inhibits the activation of glial cells in the spinal dorsal horn (Wang et al., 2018; Zhang et al., 2018; Jin et al., 2020), thus achieving remote alleviation of hyperalgesia.

The vagus nerve (VN) performs a critical function in the neuroendocrine–immune axis. The system maintains homeostasis through its afferents (the hypothalamic-pituitary-adrenal axis and the central autonomic nervous system), as well as its efferents (the cholinergic anti-inflammatory pathway) (Bencherif et al., 2011; Bonaz et al., 2013; Ohira et al., 2013). Optogenetic stimulation of PROKR2Cre-marked nerve terminals at the ST36 site can actuate the vagal-adrenal axis reflexes. The adrenal gland releases catecholamines such as dopamine to inhibit an increase in pro-inflammatory cytokines (Liu et al., 2020, 2021c). In the efferent VN-based cholinergic anti-inflammatory pathway, VN activation leads to the release of acetylcholine, which interacts with acetylcholine receptors on immune cells, resulting in lower cytokine levels (Pavlov et al., 2003; Pavlov and Tracey, 2006). With the exception of adenosine and VN hypotheses, other hypotheses include remote anti-inflammatory and analgesic effects, such as diffuse noxious inhibitory control hypothesis (Fleckenstein, 2013), segmental nerve hypothesis (Baeumler et al., 2015), and gate control hypothesis (suppressing the transmission of thin fibers by activating thick fibers) (Melzack and Wall, 1984), which may be the basis of the body points in acupuncture for the treatment of migraines.

### Limitations and perspective

Acupuncture prophylaxis for migraines is likely to be as effective as prophylactic medication. However, there are a few limitations in current clinical acupuncture literature. First, many studies used placebo acupuncture as a control. Although verum acupuncture is more effective than placebo, any skin-penetration intervention cannot be considered an inert placebo (Streng et al., 2006; Linde et al., 2009). Nevertheless, placebo acupuncture may induce a variety of unanticipated peripheral, segmental, and cerebral physiological reactions, which prevents double-blinded randomized clinical trials (RCTs) and introduces bias into clinical acupuncture studies (Yang et al., 2011). Second, there was substantial heterogeneity among the studies in terms of the acupuncture techniques used in the interventions, selection of outcome parameters, and controls. Studies with higher methodological quality in the control group and more uniform test parameters of acupuncture in treating migraine should be conducted in the future.

## Basic study status and mechanism of acupuncture protecting from neuroinflammation and neuronal sensitization of migraine

Extensive studies have shown that neuroinflammation and neuronal sensitization are the underlying causes of migraines (Noseda and Burstein, 2013; Edvinsson et al., 2019). The trigger factors of migraine are multifaceted, including environmental factors, hormonal changes, medications, and lifestyle factors, as well as a strong component of genetics, which can contribute to neuroinflammation and lead to neuronal sensitization (Maleki et al., 2012, 2013; Schwedt et al., 2015b; Charles, 2018; Qubty and Patniyot, 2020). During an attack, one or more of these factors activate peripheral nerve endings innervating the dural vasculature (Iyengar et al., 2019). The actuation of dural meningeal afferents leads to the release of neuropeptides into the perivascular space (Pietrobon and Moskowitz, 2013; Zagami et al., 2014). Signals from perivascular primary sensory neurons result in a downstream cascade of events that causes neurogenic inflammation, including protein extravasation in the dura mater, penetration of immune cells, vasodilation, and increased blood flow. The inflammatory response is followed by prolonged activation of dural first-order trigeminovascular neurons, called peripheral sensitization. Repetitive nociceptive inputs from perivascular primary sensory neurons may additionally induce the release of neurotransmitters and neuromodulators from the central terminals of primary afferent neurons in the spinal cord and trigeminal nucleus, leading to the sensitization of trigeminocervical complex (TCC) second-order neurons and trigeminothalamic third-order neurons to brainstem and diencephalon structures, that is, central sensitization (Levy, 2012; Goadsby et al., 2017). Ultimately, the nociceptive receptive field at the TCC expands, and its input threshold for migraine activation decreases (Burstein et al., 2010).

### Characteristics of current basic studies

Among the 12 basic studies on the treatment of rodent migraine models with acupuncture (Table 1), we discovered that electrical acupuncture at 2/15 Hz (amplitude-modulated wave) was the most commonly utilized (11 studies), and only one study used MA. The most commonly selected acupoint was GB20, which was applied alone in six studies. Three studies used a combination of GB20 and GB34. Two studies used TE5 to combine GB20 and GB34. GB8 alone was applied in one study. Different methods have been applied to induce recurrent migraines. Dural electrical stimulation was the most commonly utilized (six studies), with the dura inflammatory soup injection used in three studies. Infusion of nitroglycerin, unilateral electrical stimulation of the TG, and superior sagittal sinus electrical stimulation were used once in studies as diverse choices for migraine model creation. Regarding dural electrical stimulation and dura inflammatory soup injection, the most frequently stimulated site was described as a nearby bregma (seven studies), followed by the surrounding superior sagittal sinus (three studies).

**TABLE 1:** The characteristic of current basic studies of acupuncture in the treatment of migraine.

Study	Migraine model	Intervention	Acupoints	Acupuncture parameters	Controls	Behavioral measurements	Biochemical measurements
Gao et al. (2014)	Nitroglycerin-induced migraine rats (intraperitoneally)	EA	TE5, GB34	14 Hz, 0.1–1 mA for 20min	Con1: no treatment Con2: only nitroglycerin-treated Con3: electroacupuncture at nonacupoints following Nitroglycerin-treatment	The number of head-scratching↓	Glutamates, lactic acid, lactic acid, glutamine (plasma)↓; lipids (CH_2_, CH_3_), OAc, NAc, pyruvic acid, LDL/VLDL, creatine (plasma)↑
Zhou et al. (2015)	Dural electrical stimulation (An incision 2.8–3.2 mm long was made in the anterior fontanelle retrusion of 3.2–3.4 mm)	MA	GB20	Retaining needles for 20 min	Con1: no treatment Con2: only received dural electrical stimulation Con3: received MA at GB20 followed by dural electrical stimulation	−	The activation of MLCK (middle meningeal artery)↑
Liu L. et al. (2016)	Dural electrical stimulation (4 mm anterior and 6 mm posterior to the bregma on the midline suture of parietal bone, each 1 mm in diameter)	EA	GB20	2/15Hz frequency (interrupted wave) and 0.5–1.0 mA intensity for 15 min	Con1: electrode implantation without stimulation Con2: only received dural electrical stimulation Con3: received EA at a distant non-acupuncture point (approximately 10 mm above the iliac crest) after dural electrical stimulation	Resting, freezing, grooming behavior↓; exploration behavior↑	5-HT (RVM, TNC)↑; 5-HT (plasma)↓
Pei et al. (2016)	Dural electrical stimulation (4 mm anterior and 6 mm posterior to the bregma on the midline suture of parietal bone, each 1 mm in diameter)	EA	GB20	2/15Hz frequency (interrupted wave) and 0.5–1.0 mA intensity for 15 min	Con1: electrode implantation without stimulation Con2: only received dural electrical stimulation Con3: received EA at a distant non-acupuncture point (approximately 10 mm above the iliac crest) after dural electrical stimulation	Exploratory, locomotor, eating/drinking behavior↑; freezing-like resting, grooming behavior↓	c-Fos immunoreactivity neurons (PAG, RMg, TNC)↓
Zhang H. et al. (2016)	Unilateral electrical stimulation of the trigeminal ganglion	EA	GB20, TE5	2/15Hz frequency and 1.0 mA intensity for 30 min	Con1: sham operation plus minimal acupuncture (MA) as a control intervention Con2: trigeminal ganglion electrical stimulation with MA	−	CGRP, PGE2 (Serum)↓; PPE (dura mater)↓; COX2, IL-1β protein (TG)↓; CB1 receptor (TG)↑
Zhao L. P. et al. (2017)	Dural electrical stimulation (adjacent to superior sagittal sinus)	EA	GB20	2/15Hz frequency (interrupted wave) and 0.5–1.0 mA intensity for 15 min	Con1: electrode implantation without stimulation Con2: only received dural electrical stimulation Con3: received EA at a distant non-acupuncture point (approximately 10 mm above the iliac crest) after dural electrical stimulation	MWT: hind paw and facial withdrawal thresholds↑	CGRP (TG, TNC, VPM)↓
Pei et al. (2019)	Dural electrical stimulation (adjacent to superior sagittal sinus)	EA	GB20	2/15Hz frequency (interrupted wave) and 0.5–1.0 mA intensity for 15 min	Con1: electrode implantation without stimulation Con2: only received dural electrical stimulation Con3: received EA at a distant non-acupuncture point (approximately 10 mm above the iliac crest) after dural electrical stimulation	MWT: hind paw and facial withdrawal thresholds↑	5-HT7R (PAG, RMg, TNC)↓
Xu et al. (2019)	Dural electrical stimulation (adjacent to superior sagittal sinus)	EA	GB20, GB34	2/15Hz frequency (interrupted wave) and 0.5–1.0 mA intensity for 15 min	Con1: electrode implantation without stimulation Con2: only received dural electrical stimulation Con3: received EA at a distant non-acupuncture point (approximately 10 mm above the iliac crest) after dural electrical stimulation	MWT: hind paw and facial withdrawal thresholds↑; TWT: tail-flick and hot-plate latencies↑	c-Fos immunoreactivity neurons (TG)↓; CGRP, SP, VIP, PACAP, NO, ET-1 (plasma)↓; CGRP, VIP (dural)↓
Qu et al. (2020)	Dura Inflammatory soup injection (the skull was exposed 1 mm in front of the fontanelle and 1 mm left of the midline)	EA	GB8	2/15Hz frequency (interrupted wave) and 0.5–1.0 mA intensity for 15 min	Con1: dural injection 20 μL of 0.9% sterile saline Con2: dural injection of Inflammatory soup Con3: Inflammatory soup with verum MA Con4: dural injection 20 μL of 0.9% sterile saline with verum EA Con5: dural injection 20 μL of 0.9% sterile saline with verum MA	MWT: facial (periorbital region receptive field of the trigeminal nerve) withdrawal thresholds↑	WDR neuronal firings (TCC)↓
Qu et al. (2020)	Dura Inflammatory soup injection (the skull was exposed 1 mm in front of the fontanelle and 1 mm left of the midline)	EA	GB20	2/15Hz frequency (interrupted wave) and 0.5–1.0 mA intensity for 15 min	Con1: dural injection 20 μL of 0.9% sterile saline Con2: dural injection of Inflammatory soup Con3: Inflammatory soup with sham EA	MWT: facial (C1 spinal dorsal horn neurons) withdrawal thresholds↑	Spontaneous discharges of neurons (TCC)↓
Zhao et al. (2020)	Dural electrical stimulation (superior sagittal sinus)	EA	Acu1: GB20; Acu2: GB20, GB34	2/15Hz frequency (interrupted wave) and 0.5–1.0 mA intensity for 15 min	Con1: electrode implantation without stimulation Con2: only received dural electrical stimulation Con3: received EA at a distant non-acupuncture point (approximately 10 mm above the iliac crest) after dural electrical stimulation	MWT: facial withdrawal thresholds↑	Mast cell, macrophage (dural)↓; IL-1β, IL-6, TNF-α, COX-2, CGRP, BDNF (Serum)↓
Liu et al. (2021a)	Dura Inflammatory soup injection (1 mm left of midline, 1 mm anterior to bregma)	EA	GB20, GB34	2/15Hz frequency (interrupted wave) and 1.0 mA intensity for 15 min	Con1: repeated dural injection of artificial cerebrospinal fluid Con2: repeated dural injection of Inflammatory soup Con3: Inflammatory soup with sham EA	MWT: paw and facial withdrawal thresholds↑; TWT: tail-flick latency, hot-plate latency, cold-plate behaviors↑	CGRP (TG, TNC)↓; WDR neuronal firings (TNC)↓; 5-HT^7^R mRNA, c-Fos immunoreactivity neurons, PKA-p, p-ERK1/2 (TG, TNC)↓

EA, electroacupuncture; TE, triple energizer meridian; GB, governor vessel; LDL, low-density lipoprotein; OAc, O-acetyl glycoproteins; NAc, N-acetyl glycoproteins; VLDL, very-low-density lipoprotein; 3-HB, 3-hydroxybutyric acid; MA, manual acupuncture; MLCK, myosin light chain kinase; 5-HT, 5-hydroxytryptamine; RVM, rostroventromedial medulla; TNC, trigeminal nucleus caudalis; PAG, periaqueductal gray; RMg, raphe magnus nucleus; CGRP, calcitonin gene-related peptide; PGE2, prostaglandin E2; TG, trigeminal ganglion; PPE, plasma protein extravasation; COX2, cyclooxygenase-2; MWT, mechanical withdrawal threshold; VPM, ventro-posterior medial thalamic nucleus; 5-HT7R, 5-hydroxytryptamine^7^ receptor; TWT, thermal withdrawal threshold; SP, substance P; VIP, vasoactive intestinal peptide; PACAP, pituitary adenylate cyclase-activating polypeptide; NO, nitric oxide; ET-1, endothelin-1; WDR, wide dynamic range; TCC, trigeminocervical complex; IL-1β, interleukin-β; CB1, cannabinoid 1; IL-6, interleukin-6; TNFα, tumor necrosis factor-α; COX-2, cyclooxygenase-2; BDNF, brain-derived neurotrophic factor; mRNA, messenger RNA; PKA-p, protein kinase A protein; p-ERK1/2, extracellular signal-regulated kinase1/2 protein.

**FIGURE 3:**
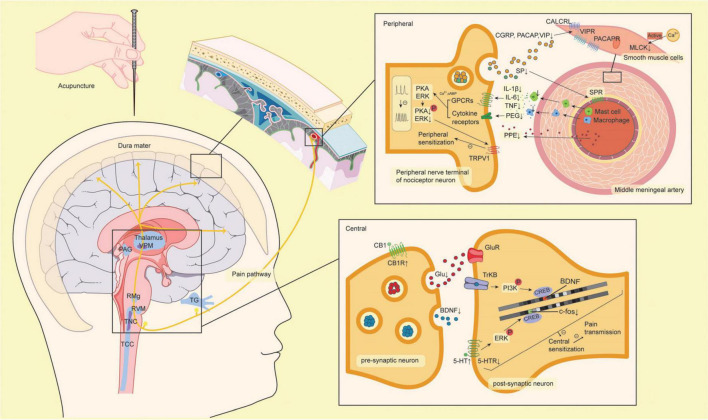
The mechanism of acupuncture protecting from neuroinflammation and neuronal sensitization of migraine. The yellow line through the brain represents the pain pathway. The factors are shown with up and down arrows, which represent up- or down-regulation from acupuncture. The pathways with minus on the line are inhibited by acupuncture. 5-HT, 5-hydroxytryptamine; 5-HTR, 5-hydroxytryptamine receptor; BDNF, brain-derived neurotrophic factor; TrkB, tropomyosin kinase B; CB1, cannabinoid 1; CB1R, cannabinoid 1 receptor; CGRP, calcitonin gene-related peptide; CALCRL, calcitonin receptor-like receptor; COX2, cyclooxygenase-2; CREB, cyclic adenosine monophosphate response element binding protein; ERK, extracellular signal-regulated kinase; Glu, glutamate; GluR, glutamate receptor; GPCRs, G protein-coupled receptors; IL-1β, interleukin-1β; IL-6, interleukin-6; MLCK, myosin light chain kinase; PACAP, pituitary adenylate cyclase-activating polypeptide; PACAPR, pituitary adenylate cyclase-activating polypeptide receptor; PAG, periaqueductal gray; PGE2, prostaglandin E2; PI3K, phosphoinositide 3-kinase; PKA, protein kinase; PPE, plasma protein extravasation; RMg, raphe magnus nucleus; RVM, rostroventromedial medulla; SP, substance P; SPR, substance P receptor; TCC, trigeminocervical complex; TG, trigeminal ganglion; TNC, trigeminal nucleus caudalis; TNFα, tumor necrosis factor α; TRPV1, transient receptor potential vanilloid 1; VIP, vasoactive intestinal peptide; VIPR, vasoactive intestinal peptide receptor; VPM, ventro-posterior medial thalamic nucleus.

Except for the acupuncture parameters and modeling methods, the measuring outcomes of migraine-related symptoms varied. Migraine pain is commonly evaluated by observing animal behavioral alterations, including spontaneous behavioral alterations (increased grooming, resting, freezing, eye blinking, and head shake and decreased locomotion, rearing, exploration, eating, and drinking), and artificially induced pain response (tactile and thermal hypersensitivity measures) (Vuralli et al., 2019). Three studies found that acupuncture reduced headache-related spontaneous behaviors in migraine models. Seven studies showed that acupuncture reduces the evoked hyperalgesia response in migraine models, as assessed by the mechanical withdrawal threshold and thermal withdrawal threshold. Overall, it is suggested that acupuncture may work by blocking pain-related brain areas from neurogenic inflammation, thus modulating neuronal sensitization for migraine treatment, which we have outlined in detail below (Figure 3).

### Neuroinflammation

Inflammation is an intricate physiological reaction of the somatosensory, immune, neuronal, and vascular/circulatory systems to tissue injury, infection, or irritants. The basic studies we included shows that acupuncture could reduce neuroinflammation by reducing the release of trigeminal-activated neuropeptides, inhibiting dural immune cells, and downmodulating inflammatory mediator levels, which are detailed discussion below.

#### Trigeminal-activate neuropeptides release

Neuropeptides participate in trigeminal activation, which is believed to be a crucial stage in the processing of migraine pain, including calcitonin gene-related peptide (CGRP), substance P (SP), and pituitary adenylate cyclase-activating polypeptide (PACAP) (Pietrobon and Moskowitz, 2013; Zagami et al., 2014; Iyengar et al., 2019). With peripheral nerve action, trigeminal nociceptors mediate the release of CGRP, SP, and PACAP into perivascular spaces, leading to the development of inflammation (Kaiser and Russo, 2013; Kursun et al., 2021). These compounds influence the trigeminovascular pathway and dural meningeal nociceptive function by increasing plasma extravasation, widening cranial blood vessels, and stimulating sensory nerve transmission (Moskowitz, 1993; Edvinsson, 2004; Ghzili et al., 2008; Csati et al., 2012; Rudecki and Gray, 2016). According to current studies, acupuncture may prevent elevated CGRP, SP, and PACAP levels. Xu et al. (2019) demonstrated that acupuncture at GB20 and GB34 downregulated CGRP, SP, and PACAP in plasma.

Acupuncture has also been demonstrated to reduce CGRP in serum (Zhang H. et al., 2016; Zhao et al., 2020) and migraine-related brain areas including the TG, TNC, ventroposterior medial thalamic nucleus, and dural (Zhao L. P. et al., 2017; Xu et al., 2019; Liu et al., 2021a). CGRP is the most abundant neuropeptide in trigeminal sensory nerve stores (Williamson and Hargreaves, 2001). The pivotal contribution of CGRP in migraine pathophysiology comes from the development of targeted drugs against CGRP or its receptor. Because of the difficulty in overcoming side effects, including hypersensitivity reactions, cardiovascular disease, and cerebrovascular disease (Ashina et al., 2021), more clinical studies are required to make acupuncture an alternative strategy to target CGRP.

#### Dural immune cells activate

The dura mater contains extracerebral blood vessels, fibroblasts, trigeminal afferent endings, and numerous immune cells (Erdener et al., 2021). After peripheral nerve action and the release of CGRP, SP, and PACAP, macrophages may become activated, and mast cells degranulate (Manning et al., 2016; Zhou et al., 2022). The production of inflammatory substances during degranulation, including histamine, proteases, and cytokines, is thought to contribute to headaches (Levy et al., 2007). Zhao et al. (2020) found that acupuncture at GB20 and GB34 decreased the number of mast cells and macrophages in the dural. There is insufficient research on how acupuncture affects immune cells in the brain.

#### Inflammatory related mediators release

Activation of dural immune cells and production of potent proinflammatory chemicals. These inflammatory molecules further stimulate and sensitize meningeal nociceptors to promote migraine attacks (Baun et al., 2012; Forsythe, 2019). Zhang H. et al. (2016) observed that acupuncture induced decreases in serum prostaglandin E2 (PGE2) levels as well as interleukin-β and cyclooxygenase-2 protein expression in the TG. Zhao et al. (2020) found that interleukin-6 and tumor necrosis factor-α levels were both considerably reduced by acupuncture. Vasodilating neurotransmitters are increased in the plasma during migraine attacks and at headache-free intervals. Vasodilation is accompanied by increased flow and is a key element in inflammation, which allows inflammatory cells and plasma proteins to accumulate at the site of injury. Vasoactive intestinal peptide (Goadsby et al., 1990; Cernuda-Morollón et al., 2015), endothelin (Gallai et al., 1994), and nitric oxide (Uzar et al., 2011) levels are significantly increased in migraine attacks, and acupuncture can reduce the levels of vasoactive neurotransmitters in the plasma and dural (Xu et al., 2019).

Myosin light chain kinase is involved in vascular contraction (van Rossum et al., 1997; Gz, 1999). Zhou et al. (2015) that the activation of myosin light chain kinase in the middle meningeal artery was increased by acupuncture at GB20. Plasma protein extravasation (PPE) is a feature of neuroinflammation that represents increased vascular permeability. Zhang H. et al. (2016) demonstrated that acupuncture could alleviate electrically evoked meningeal PPE. The reduction in PPE reflects the regulation of vasoactive substances by acupuncture, which can modulate multiple inflammatory mediators and reduce inflammation in a variety of ways.

### Neuronal sensitization

Neuronal sensitization is a developmental stage of neuroinflammation. It shows up as a decrease in activation thresholds and an increase in the reactivity of nerve terminals to simulation or injury. In peripheral sensitization, the post-translational processing of intracellular signaling pathways is activated by the interaction of inflammatory molecules and their receptors (McKinnon et al., 2015). When inflammatory molecules bind to their respective receptors on peripheral nociceptor nerve fibers, second messengers are produced accordingly, which, in turn, activate selected kinases. Activated kinases cause nociceptor neurons to become hypersensitive and hyperexcitable by lowering the ion channel threshold, increasing membrane excitability, or boosting receptor expression (McKinnon et al., 2015). Central sensitization is initiated by peripheral sensitization and is characterized by an improvement in the functional of neurons and circuits in nociceptive pathways across the neuraxis, resulting from the increase in membrane excitability, synaptic efficacy, or a decrease in inhibition (Latremoliere and Woolf, 2009). Cytokines affect neuroplasticity by acting as regulatory mediators in the development of central sensitization. It has been shown in studies below that acupuncture can reduce neuronal sensitization in migraines by inhibiting related kinases, reducing cytokine levels, and relieving neuronal activation in migraine-related brain areas. In addition, several neuromodulatory systems are also involved in the process of neuronal sensitization in migraine, including the endocannabinoid and serotonin systems.

#### Related mediator release

Brain-derived neurotrophic factor (BDNF) is one of the most prevalent neurotrophins in the brain and is upregulated by inflammatory activation relevant factors (Marini et al., 2004; Lipsky and Marini, 2007; Xu et al., 2007). A substantial amount of evidence relates BDNF to neuronal maintenance and survival, plasticity, and neurotransmitter modulation and has been recognized as an important modulator of the enhancement of synaptic efficacy in central and peripheral nociceptive pathways (Mannion et al., 1999; Thompson et al., 1999; Lu et al., 2014). Zhao et al. (2020) observed that acupuncture at GB20 and GB34 inhibited serum BDNF levels. Glutamate is an excitatory amino acid neurotransmitter in the mammalian central nervous system (CNS). In prior *in vitro* trials, local glutamate has been shown to activate receptors on local osteocytes, chondrocytes, and synoviocytes to exacerbate local pathologies (Lawand et al., 1997; Laketić-Ljubojević et al., 1999; Gu et al., 2002; Flood et al., 2007; Ramage et al., 2008; McNearney et al., 2010). Glutamate was observed to be increased in plasma from migraine model rats and downregulated by acupuncture (Gao et al., 2014). Acupuncture may provide additional potential benefits in neuronal sensitization by moderating neurotrophins, and neurotransmitters, highlighting neuronal-sensitization-related mediators as a novel area for research on acupuncture in treating migraine.

#### Increased neural activity

An increase in neural activity is a direct sign of neuronal sensitization. Numerous studies have observed the enhancement of neural activity in pain-related brain areas of migraine by recording spontaneous discharges of neurons, WDR neurons, and c-Fos immunoreactive neurons (Sandkühler, 2009). WDR neurons were identified based on their enhanced responses to mechanical stimulation from non-noxious to noxious receptive fields. Previous research has shown that the mechanisms underlying hyperalgesia/allodynia (Takeda et al., 2012; Shimazu et al., 2016; Takehana et al., 2017) and migraine attacks (Storer et al., 2004; Hou and Yu, 2010) involve WDR neurons in pain-related areas. c-Fos is a commonly used protein marker of neuronal activity (Harris, 1998). Studies have demonstrated that acupuncture can decrease spontaneous and WDR neuronal discharges in TCC-Fos neurons in the periaqueductal grey (PAG), raphe magnus nucleus, TNC, and TG (Pei et al., 2016; Xu et al., 2019; Liu et al., 2021a). Notably, Qu et al. (2020) found that acupuncture can rapidly reduce C-fiber-triggered WDR neuronal firing of TCC within 60 s, indicating the potential of acupuncture as an essential supplemental and alternative strategy for migraine patients who cannot respond to acute medicine.

#### Endocannabinoid system modulation

The endocannabinoid system is a ubiquitous neuromodulatory network that participates in both the development of the CNS and modulation of neuronal activity and network function (Lu and Mackie, 2021). Cannabinoid 1 (CB1) receptors are primary CNS receptors and are highly expressed in a number of brain and supraspinal regions that are involved in nociceptive transmission in neurons (Barrie and Manolios, 2017). CB1 receptors are abundant in synaptic terminals (Nyíri et al., 2005). Zhang H. et al. (2016) observed that CB1 receptors appear to mediate anti-inflammatory effects, and acupuncture can induce CB1 receptor expression in TG. Cannabinoid pharmacology has been explored for pain management. However, a variety of psychotropic side effects make its clinical application ineffective (Fitzcharles et al., 2012), making acupuncture a potential alternative therapy for regulating the endocannabinoid system.

#### Serotonin system modulation

Both peripheral and central serotonin systems have been demonstrated to play crucial roles in migraine pathogenesis through their impact on trigeminovascular nociceptive information transmission and central sensitization (Hamel, 2007). Liu L. et al. (2016) found that acupuncture attenuated peripheral 5-hydroxytryptamine (5-HT) release and increased central 5-HT levels in the rostroventromedial medulla (RVM) and TNC. 5-HT may play a different role depending on the receptor. The 5-HT7 receptor subtype (5-HT7R), has been shown to modulate signals along the descending pain pathway and mediate central sensitization in migraine (Wang et al., 2010; Ramírez Rosas et al., 2013). A decrease in 5-HT7R expression has been observed in the PAG, raphe magnus nucleus, TNC, and 5-HT7R messenger RNA expression in the TG and TNC after acupuncture (Pei et al., 2019; Liu et al., 2021a). Studies above have shown different effects of acupuncture on the central serotonin system, which may be due to differences in experimental conditions, measurement targets, or random false positives.

### Limitations and perspective

Neuroinflammation and neuronal sensitization are complex processes that involve the coordination of multiple cells, systems, and feedback cycles. There is a strong interplay between neuroinflammation and neuronal sensitization. Neuroinflammation causes neuronal sensitization, which is involved in and further aggravates neuroinflammation through the interplay between different molecular signaling pathways (Ji et al., 2018). As for peripheral sensitization, nociceptors and immune cell interactions are reciprocal, to rapidly control resident immune cells and draw circulating cells to the site of local inflammation. Nociceptors release cytokines and chemokines, which activate primary afferents and cell bodies in the nerve (Zhang et al., 2013; Liu X. J. et al., 2016). Central sensitization can modulate glia-specific receptors and channels, such as the downregulation of glutamate transporters (glutamate transporter 1 and glutamate aspartate transporter) in spinal cord astrocytes, which causes glutamate accumulation in synaptic clefts and results in neuronal hyperactivity (Sung et al., 2003; Ji et al., 2013).

Central sensitization could also manifest as “leaky” astrocytes, causes the increase of secreting cytokines and chemokines (Ren and Torres, 2009; Chen G. et al., 2014). Therefore, neuronal sensitization and neuroinflammation together lead to recurrent migraines. Basic studies have provided evidence for the usefulness of acupuncture in neuroinflammation and neuronal sensitization-driven migraine in different ways. However, the results are incomplete; they did not account for the complex integration of the neuroimmune network. Moreover, the sample size in all studies were insufficient which may increase the possibility of false positives. Future studies should systematize the effects of acupuncture on neuroinflammation and neuronal sensitization using adequate sample sizes.

Furthermore, there are some deficiencies due to the limitations of technology in basic studies. Currently, there is no unified standard for acupoint positioning in animals (Cheng et al., 2021). The lack of uniform and standardized acupoint positioning methods may result in the bias in basic studies of acupuncture. There are also doubts about the translation of human acupoints to animals (Cl et al., 2016), which requires more exploration and discussion. Current animal models all produce acute headache conditions, which present an acute inflammatory response and transient hyperalgesia, both of which diminish with time. Research has focused only on the effectiveness of acupuncture in the initial stages of neuroinflammation and neuronal sensitization. Better models are required to investigate the long-term efficacy of acupuncture against neuroinflammation and neuronal sensitization in migraines.

## Brain function alterations underlying the efficacy of acupuncture treatment for migraine

### Characteristics of current neuroimaging studies

#### Participants

A total of 610 patients and 469 controls (sham acupuncture and healthy controls [HCs]) were included in this review of 16 trials. Eleven studies enrolled patients who had MwoA. Three studies did not define migraine subtypes, one study enrolled patients with CM, and one study enrolled patients with menstrual migraines. Eleven studies applied the self-control model, and five explored the differences in cerebral responses between verum acupuncture and sham acupuncture.

#### Acupuncture intervention

Fifteen trials applied MA, and one applied EA. Thirty acupoints were selected for the studies. SJ5 was most commonly used, followed by GB34 and GB20. Eight of the top 11 most frequently used points (≥5 times) were from the Shaoyang meridian. Fourteen trials reported acquisition of the Deqi sensation, and 13 reported depth of insertion. The duration of treatment varied from 8 to 30 min for one to 36 weeks and mostly applied five sessions per week.

#### Trial design and data analysis

There were six RCTs and ten controlled trials. Nine trials showed significant clinical results after acupuncture. Eight studies used the VAS and showed significantly decreased pain intensity in migraine. In addition, migraine-related aspects, such as the frequency of migraine attacks, migraine attack duration, and headache days, also had clinical improvements. One study reported relief with the measure of MSQ in life quality, and three with a self-rating anxiety scale and a self-rating depression scale to assess the anxiety and depression aspects of migraineurs. Eleven studies reported correlations between functional neurological alterations and altered clinical outcomes due to acupuncture.

Twelve studies selected resting-state functional magnetic resonance imaging (rsfMRI) to investigate cerebral neurological responses through acupuncture in migraine patients, among which one applied diffusion tensor imaging. Two studies applied positron emission tomography-computed tomography, one selected proton magnetic resonance spectroscopy, and one applied functional transcranial Doppler focusing on the alteration of cerebral blood flow velocity in the cerebral arteries. Of the functional magnetic resonance imaging (fMRI) trials, three applied independent component analysis (ICA) and two combined with seed-based analysis (SBA), one used machine learning, three used regional homogeneity (ReHo) analysis, and three used an amplitude of low frequency (ALFF) analysis. A total of 13 trials focused on the sustained impact after long-term acupuncture treatment; three studies explored the immediate effects of a single treatment with acupuncture. The detailed features of the neuroimaging studies are summarized in Table 2.

**TABLE 2:** The neuroimaging characteristic of current trials of acupuncture in the treatment of migraine.

Study	Clinical trial design	Clinical condition	Intervention	Comparison	Neuroimaging technology	Acupoints	Acupuncture parameters	Outcomes
Wei et al. (2022)	Pilot study	MwoA	EA (*n* = 30)	HC (*n* = 30)	rsfMRI	GB8	15° angle, 0.5 inches, 8 min, once EA: 2 Hz, 1 mA	**FC:** Right dorsal anterior insula-right postcentral gyrus↓ Right posterior insula-left precuneus↑ **Correlation:** FC value of the left precuneus was positively correlated with VAS score
Zhang et al. (2021)	RCT	MMoA	Acupuncture (*n* = 25)	Sham acupuncture (*n* = 25)	rsfMRI (ALFF, ReHo)	Bilateral GB20, GB8, PC6, SP6, LR3	differed in 0.5–1.5 cm, twisted and rotated (90°< amplitude <180°) 1–2 Hz, deqi, 30 min, 3 months, 9 ± 2 times a month	**Clinical outcome:** SAS, SDS, VAS, intensity of migraine, frequency of attacks ↓ **ALFF:** Right middle frontal gyrus↑ Left anterior cingulate, right inferior frontal gyrus↓ **ReHo:** Right superior frontal gyrus, left cuneus, and right MFG↑ Right superior temporal gyrus↓ **Correlation:** Altered ALFF value in the left ACC was positively correlated with the decreases in the SAS, SDS scores in TA
Tian et al. (2021)	Controlled trial	MwoA	Acupuncture (*n* = 52)	HC (*n* = 60)	rsfMRI	Bilateral GB34, GB40, TE5, GB33, GB42, TE8, ST36, ST42, LI6	5–15 mm, deqi, 30 min, 4 weeks, 5 sessions per week	**Clinical outcome:** SAS, SDS, headache intensity↓ **FC:** Amygdala and insula, Amyg and SFG, CG and SFG, Hipp and SFG, and Tha subregions↓ Amygdala and MFG, Hipp and MFG, Hipp and INS, IPL and INS, IPL and MFG, IPL and SFG, and Tha subregions↑ **Correlation:** FCs between the left Amyg and left MFG, the left Amyg and left SFG correlated with the rate of improvement in headache intensity
Liu et al. (2021b)	Controlled trial	MwoA	Acupuncture (*n* = 40)	HC (*n* = 16)	rsfMRI (ReHo)	GV20, EX-HN5, GB5, GB15, LI4, LR3 EA: bilateral GB20, GB8,	Deqi, 20 min, 6 weeks, 2 sessions per week EA: 2 Hz, 0.1–1.0 mA	**Clinical outcome:** Migraine days, VAS, SAS, SDS↓ MSQ↑ **ReHo:** Cerebellum, angular gyrus↑ (after 12 acupuncture sessions) **Correlation:** Positive correlations of the change in ReHo value in the angular gyrus with days of migraine at baseline and with the change in number of days with migraine
Yin et al. (2020)	Controlled trial	MwoA	Acupuncture (*n* = 40)	HC (*n* = 40)	rsfMRI (zALFF)	Bilateral TE5, GB34, GB40, TE8, GB33, GB42, LI6, ST36, ST42	Deqi, 30 min, 4 weeks, 5 sessions per week	**Clinical outcome:** VAS↓ **zALFF:** Right middle occipital gyrus and left middle occipital gyrus↓ **Correlation:** zALFF value of right middle occipital gyrus positively correlated with the improvement of VAS scores zALFF value of left middle occipital gyrus positively correlated with the relief of mean migraine days zALFF value of right fusiform positively correlated with the duration
Zou et al. (2019)	Controlled trial	CM	Acupuncture (*n* = 14)	HC (*n* = 18) Self-control (*n* = 14)	rsfMRI (ICA,SBA)	Bilateral: TE5, GB20, GB8, ST8	5–15 mm, deqi, 30 min, 36 weeks, 3 sessions per week	**Clinical outcome:** Headache attacks, monthly mean/immediate VAS, headache days, acute headache medications↓ **FC:** Between the right temporal lobe and left ACC, between the right TPL and bilateral superior medial gyrus, and between the right TPL and right PRECUN↑ **Correlation:** Increased z-scores within the DMN (L_SPFG and L_PRECUN) were associated with reduced immediate VAS scores, and increases in z-scores of the L_PRECUN were negatively correlated with reductions in the monthly amount of acute headache medications.
Gu et al. (2018)	Controlled trial	MwoA	Acupuncture (*n* = 15)	HC (sham acupuncture) (*n* = 14)	MRS	GV20; bilateral GB20, LR2	1.5–2.5 cm, deqi, EA with dilatational wave, 30 min, 5 sessions, once per week	**Clinical outcome:** VAS, mean duration of headache attacks↓ NAA/Cr in the bilateral thalamus↑ **Correlation:** Correlation between NAA/Cr and VAS in bilateral thalamus in post-treatment follow-up
Li et al. (2017)	Controlled trial	MwoA	Acupuncture (*n* = 100) VA (*n* = 60) SA (*n* = 20) WT (*n* = 20)	HC (*n* = 46)	rsfMRI (ALFF)	Bilateral VA1: GB34, GB40, TE5 VA2: GB33, GB42, TE8 VA3: ST36, ST42, L16	5–15 mm, deqi, 30 min, 4 weeks, 5 sessions per week	**ALFF:** **Post- vs. pre-treatment:** Bilateral orbitofrontal cortex, bilateral RVM/TCC and bilateral rostral midbrain↑ Left middle occipital cortex/cuneus↓ **VA vs. SA:** Bilateral RVM/TCC↑
Li et al. (2017)	Controlled trial	MwoA	Acupuncture (*n* = 100) VA (*n* = 60) SA (*n* = 20) WT (*n* = 20)	HC (*n* = 46)	rsfMRI ICA.SBA	Bilateral VA1: GB34, GB40, TE5 VA2: GB33, GB42, TE8 VA3: ST36, ST42, L16	5–15 mm, deqi, 30 min, 4 weeks, 5 sessions per week	**ICA outcomes:** **Post- vs. Pre-treatment** rs-fc of the right precuneus with rFPN, of left middle frontal gyrus with rFPN↓; rs-fc with the bilateral posterior cingulate cortex for rFPN↑ Right precuneus rs-fc with the bilateral rACC/mPFC, ventral striatum, middle/inferior occipital gyrus, cuneus, DLPFC and cerebellum, left VLPFC and right superior temporal gyrus↑ **Correlation:** Decreased rs-fc with the bilateral precuneus, right paracentral gyrus and postcentral gyrus for rFPN positively associated with a decrease in headache intensity
Zhang Y. et al. (2016)	controlled trial	MwoA	Acupuncture (*n* = 12)	HC (*n* = 12)	rsfMRI	Bilateral: TE23, GB8, GB20, EX-HN5, LI4, LR3, TE5, GB34, GB41	1.5–2.5 cm, deqi, 30 min, 4 weeks, 5 sessions per week	**Clinical outcome:** VAS and PSQI scores, duration and frequency of migraine attacks↓ **FC:** Bilateral superior frontal gyrus, medial frontal gyrus, precuneus, inferior parietal lobule, posterior cingulate cortex, cingulate gyrus, superior temporal gyrus, middle temporal gyrus, and supramarginal gyrus↑
Li et al. (2016)	controlled trial	MwoA	Acupuncture (*n* = 100) VA (*n* = 60) SA (*n* = 20) WT (*n* = 20)	HC (*n* = 46)	rsfMRI SBA	Bilateral VA1: GB34, GB40, TE5 VA2: GB33, GB42, TE8 VA3: ST36, ST42, L16	5–15 mm, deqi, 30 min, 4 weeks, 5 sessions per week	**FC:** **Post- vs. Pre-treatment:** FC between vlPAG and the bilateral middle cingulate cortex and rACC, and left mPFC↑ **VA vs. SA:** FC between vlPAG and the bilateral middle cingulate cortex and rACC, and left mPFC↑ **Correlation:** Negative association between VAS (post-pre) and rs-fc (post-pre) between vlPAG and brain regions including bilateral rACC and MCC, and left superior frontal gyrus, thalamus, putamen, caudate and cerebellum, and right supplementary motor area (SMA)/preSMA and middle frontal gyrus
Li et al. (2015)	Controlled trial	MwoA	Acupuncture (*n* = 12)	HC (*n* = 12)	rsfMRI (ICA) DTI	Bilateral: TE23, GB8, GB20, EX-HN5, LI4, LR3, TE5, GB34, GB41	1.5–2.5 cm, deqi, 30 min, 4 weeks, 5 sessions per week	**Clinical outcome:** VAS scores, duration and frequency of migraine attacks↓ **FC:** FC with the RFPN in the left precentral gyrus, the left supramarginal gyrus, the left inferior parietal lobule, and the left postcentral gyrus↑ **Correlation:** Increased FC was negatively correlated with the decrease of VAS scores after treatment
Zhao et al. (2014)	RCT	MwoA	Acupuncture (*n* = 40)	Inactive acupuncture (*n* = 40)	rsfMRI (ReHo)	Group A: bilateral TE5, GB20, GB34, GB40; Group B: bilateral TE22, PC7, GB37, SP3	2.5–3.5 cm, rotation (90°< amplitude <180°) at a frequency of 1–2 Hz repeated 1–3 times to acquire deqi, 30 min, 8 weeks, 4 sessions per week	**Clinical outcome:** VAS↓ **ReHo:** ReHo in the bilateral thalamus, ACC, STG, SMA, insular, cuneus, lingual gyrus, cerebellum, and brainstem ↑ ReHo in the bilateral posterior cingulate cortex (PCC), middle frontal gyrus (MFG), angular gyrus, precuneus, middle temporal gyrus (MTG), left hippocampus, inferior parietal lobule, inferior temporal gyrus (ITG), and right postcentral gyrus↓ **Correlation:** Decrease in the VAS score was significantly related to the increased average ReHo values in the ACC
Yang et al. (2014)	RCT	Migraine	Acupuncture (*n* = 10)	Sham acupuncture (*n* = 10) Migraine group (blank) (*n* = 10)	PET-CT	Bilateral: TE19, TE8, GB33	15–30 mm, deqi, electrodes on auxiliary needles punctured 2 mm lateral to the points and 2 mm in depth, 100 Hz, 0.1–1.0 mA, 30 min, once	Bilateral middle frontal gyrus, left postcentral gyrus, left precuneus, right parahippocampus, left cerebellum and left middle cingulate cortex↑ Left Middle Temporal Cortex (MTC)↓
Yang et al. (2012)	RCT	Migraine	Acupuncture (*n* = 10)	Control acupuncture group (*n* = 10) Migraine group (blank) (*n* = 10)	PET-CT	Bilateral: TE5, GB34, GB20	EA: 15–30 mm, deqi, electrodes on auxiliary needles punctured 2 mm lateral to the points and 2 mm in depth, 100 Hz, 0.1–1.0 mA, 30 min, once	Brain metabolism in the middle temporal cortex (MTC), orbital frontal cortex (OFC), insula, middle frontal gyrus, angular gyrus, post-cingulate cortex (PCC), the precuneus, and the middle cingulate cortex↑ Brain metabolism in the parahippocampus, hippocampus, fusiform gyrus, postcentral gyrus, and cerebellum↓
Bäcker et al. (2004)	Pilot study	Migraine	Acupuncture (*n* = 10)	HC (*n* = 10)	Functional Transcranial Doppler	GB41, LR3, TE5, EX-HN5, TE23, GV20, GB20	GB41, LR3 30 min, 10 sessions, twice a week for the first 4 weeks and once a week for the following 2 weeks	Overshooting cerebral blood flow velocity and a delayed decline in MCA, PCA.

MMoA, menstrual migraine without aura; EM, episodic migraine; CM, chronic migraine; MwoA, migraine without aura; HC, healthy controls; VA, verum acupuncture; SA, sham acupuncture; WT, waiting-list; SAS, self-rating anxiety scale; SDS, self-rating depression scale; ICA, independent component analysis; VAS, Visual Analog Scale; MIDAS, Migraine Disability Assessment scale; rp, responders; nrp, non-responders; NAA, N-acetylaspartate; Cr, creatine; PPT, pressure pain threshold; BoNTA, Onabotulinumtoxin A; rACC, rostral anterior cingulate cortex; vlPAG, ventrolateral periaqueductal gray; mPFC, medial prefrontal cortex; STG, superior temporal gyrus; SMA, supplementary motor area; SFG, superior frontal gyrus; MFG, middle frontal gyrus; MCC, middle cingulate cortex; ReHo, regional homogeneity; CBFV, cerebral blood flow velocity; PCA, posterior cerebral artery; MCA, middle cerebral artery; TENS, transcutaneous electrical nerve stimulation; NS, no significant; EA, electrical acupuncture; FC, functional connection; ALFF, amplitude of low frequency; rsfMRI, resting state functional magnetic resonance imaging; CG, cingular gyrus; SBA, seed-based analysis; RVM, rostroventromedial medulla; TCC, trigeminocervical complex; RCT, randomized clinical trial; DTI, diffusion tensor imaging; PET-CT, positron emission tomography-computed tomography; MRS, magnetic resonance spectroscopy; MSQ, migraine-specific quality of life questionnaire; PFC, prefrontal cortex; FPN, fronto-parietal network; DLPFC, dorsolateral prefrontal cortex; VLPFC, ventrolateral prefrontal cortex; IPL, inferior parietal lobe; GB, gallbladder meridian; ST, stomach meridian; LI, large intestine meridian; GV, governor vessel; EX-HN, Tojingbu Xue Points of Head and Neck; LR, liver meridian; SP, spleen meridian; PC, pericardium meridia; TE, triple energizer meridian.

### Neural mechanisms underlying acupuncture analgesia

#### Descending pain modulatory system

The DPMS, which mainly contains the PAG and its descending projections to the RVM, contributes to the endogenous opioid system for pain relief. Likewise, animal studies have shown that the descending regulation of TCC *via* the ventrolateral periaqueductal gray (vlPAG) and RVM might activate “on” cells and inhibit “off” cells in the RVM, which appears to be critical for TCC activation and, consequently, migraine headache formation (Moreau and Fields, 1986; Porreca et al., 2002; Akerman et al., 2011). Our following paragraph aimed at summarizing the functional alterations after acupuncture in the PAG and RVM, which may offer evidence to the acupuncture analgesia effects under the potential DPMS pathway.

##### Periaqueductal gray

Studies have shown that the PAG, which is regarded as a main region in DPMS, is implicated in the regulation and expression of anxiety, fear, and pain, and is intimately related to opioid analgesia (Behbehani, 1995). Disrupted homeostasis of the trigeminovascular nociceptive pathway has been suggested to be a critical element in migraine headache sensitivity. Disrupted homeostasis in activity has been found to exist between the vlPAG regulating antinociception and vascular control (Li et al., 2016). Li et al. reported diminished functional connectivities (FCs) of the PAG with other DPMS areas, including the rostral anterior cingulate cortex (rACC) and the medial prefrontal cortex (mPFC) in MwoA patients, normalized after a 4-week acupuncture treatment. The normalized FC between the PAG and rACC showed significant associations with improvement in headache intensity after acupuncture (Li et al., 2016). These results are consistent with similar results of reduced FC in the PAG and its correlation with increased migraine frequency (Mainero et al., 2011). Furthermore, since the rACC/mPFC seems to be a rich opioid receptor region and a major component of the endogenous opioid system, the findings above offer evidence to the hypothesis that acupuncture may alleviate migraine symptoms through endogenous opioid DPMS. Moreover, the PAG contains longitudinally oriented columns that produce defensive behaviors combined with powerful analgesic effects when stimulated (Keay and Bandler, 2002). In other words, acupuncture may affect the activation of the PAG, thus altering other related DPMS areas as the neural pathophysiology of migraine analgesia.

##### Rostroventromedial medulla and trigeminocervical complex

The DPMS includes the RVM and TCC, two well-known regions implicated in nociceptive perception and migraine pathophysiology (Millan, 2002). The descending modulation of TCC signals starts primarily from the PAG and RVM (Akerman et al., 2011). An increasing number of human brain neuroimaging studies in migraine patients support the descending modulation (RVM, TCC) dysfunction hypothesis based on animal models (Stankewitz and May, 2011; Stankewitz et al., 2011; Kröger and May, 2015). A clinical trial conducted by Li et al. showed that MwoA patients exhibited lower ALFF values in RVM/TCC. Long-term verum acupuncture was able to normalize the decreased ALFF, which is in line with the acupuncture effects on other regions of the DPMS and in other chronic pain conditions (Chen X. et al., 2014; Egorova et al., 2015). Other studies have suggested that the inhibitory effects of the neuropeptide CGRP were discovered in certain TCC subregions (Covasala et al., 2012). CGRP downregulation after acupuncture has been reported in migraine, which may support the critical role of acupuncture effects in TCC in the central and peripheral pathways relieving migraine attacks. These recent studies have already shown that verum acupuncture is effective in normalizing the impaired DPMS regions (e.g., PAG, RVM, and TCC) in migraine. However, more conceptual replication work should be done, and further research may focus on their correlations with other subcortical areas (Chen X. et al., 2014; Chen et al., 2015).

#### Default mode network

The DMN is a constellation of subcortical areas and appears to be implicated in higher-order cognitive functions as well as the perception of painful stimuli, since it has been revealed to have drastic changes in chronic pain disorders (Baliki et al., 2008; Raichle, 2015; Kuner and Flor, 2016). A number of studies have shown disrupted DMN connectivity in migraine patients during the interictal period (Xue et al., 2012), including in the anterior cingulate cortex (ACC) (Zhao et al., 2013), prefrontal, and temporal regions (Tessitore et al., 2013). Studies also showed that verum acupuncture analgesia may be achieved by modulating resting-state brain function and modifying the functional connections between the DMN and related brain regions (Li et al., 2017; Zou et al., 2019; Wei et al., 2022).

##### Anterior cingulate cortex

Anterior cingulate cortex is regarded as part of the DMN and is considered to be implicated in emotional functions, encompassing cognitive-attentional (assessment of pain as unpleasant) and evaluation dimensions of migraine (Beckmann et al., 2009). Further studies indicated its critical role in pain modulation and analgesia, both of which are mediated by the endogenous opioid system (Wager et al., 2004). Decreased ALFF and increased ReHo values in the ACC after verum acupuncture treatment in different subtypes of migraine and showed associations with decreased self-reported anxiety, self-reporting depression, and VAS score (Zhao et al., 2014; Zhang et al., 2021). Other studies have shown that compared with sham acupuncture, FC between the rACC and precuneus significantly enhanced after treatment (Li et al., 2017). The ALFF/ReHo value of vlPAG in MwoA patients increased significantly after acupuncture, which was related to the improvement of the VAS score (Li et al., 2016). These correlations jointly indicate that acupuncture is implicated in the encoding of pain sensation and nociceptive emotions through the ACC (de Tommaso et al., 2005). The above findings may provide supportive evidence for the hypotheses that acupuncture alterates the activity of the ACC, resulting in an alteration of the perception of nociceptive emotions triggered by headache pain.

##### Precuneus

The precuneus, a key node in the DMN, is responsible for self-referential processing and interception and participates in the determination of pain sensitivity and pain thresholds (Smith et al., 2009; Goffaux et al., 2014; Zhang J. et al., 2016). Prior studies have indicated that the precuneus and DMN have been implicated in headache disorders, such as migraine (Tessitore et al., 2013; Zhang J. et al., 2016). A recent study combining ICA and SBA found diminished FC between the right precuneus and the right frontoparietal network (rFPN), which correlated with decreased VAS scores in patients with MwoA. Therefore, the group conducted SBA, indicating that the precuneus FCs with key regions in the reward, cognitive control, and DPMS were significantly enhanced after treatment (Li et al., 2017). Another study reported that a diminished FC between the left precuneus and right posterior insula was found after EA treatment and correlated with a similar decline in VAS scores (Wei et al., 2022). The precuneus has been implicated in the pathogenesis of migraine and acupuncture-induced analgesia. Taken together, these results suggest that the DMN, as well as the precuneus, has the potential to be used as biomarkers for evaluating the therapeutic effect of acupuncture treatment. Therefore, further research may focus on the connectivity of the precuneus within the DMN with the cognitive control network and DPMS, which may all be involved in the modulation of analgesia-related effects of pain processing by acupuncture treatment.

##### Medial prefrontal cortex

The mPFC is a critical component of the DMN that combines inputs from multiple regions and thus plays a vital role in the emotional processing of migraine. Focusing on long-term acupuncturing effects in migraine patients, studies have reported significantly enhanced FCs between the mPFC and vlPAG/precuneus and suggested acupuncturing enhancement between the DPMS and within the self-referral DMN (Li et al., 2016, 2017). The prefrontal cortex, a high-order brain region that projects to the PAG, contributes to the reduced elaboration of nociceptive processing, mainly through the release of endogenous opioids, thus modulating the DPMS (Floyd et al., 2000; DaSilva et al., 2014). Moreover, using neuroimaging analysis assessed by Positron Emission Tomography scans with a specific radiotracer technique, Wager et al. (2007) observed activation induced by μ-opioid in placebo analgesia and connections enhanced between the prefrontal cortex and an interconnected subsystem of limbic regions. Based on these findings, we may get a glimpse of the pain relief produced by acupuncture treatment being associated with the altered activation of the high-order brain regions (DMN) and their increased connections with the descending inhibitory system. Thus, the acupuncturing effects in cognitive-related regions were consistent with the pain pathway.

#### Thalamus

It is well known that the thalamus, being part of the lateral pain system, conducts the third-order neuron in arousal, transmission, and attention pathways of pain (Sánchez del Rio and Alvarez Linera, 2004). Previous studies have highlighted that interference with bidirectional information flow between the thalamus and other subcortical areas consequently causes sensory and cognitive disturbances in the migraine process (Coppola et al., 2016; Hodkinson et al., 2016). A previous rsfMRI study reported that long-term acupuncture treatment results in substantial ReHo alterations in the bilateral thalamus in patients with migraine, including the ventral lateral posterior nucleus and ventral posterior medial (Hirai and Jones, 1989; Zhao et al., 2014). Since both nuclei are considered to be involved in the processing of spatial and intensity aspects of noxious stimuli, we can speculate that acupuncture directly regulates these nuclei involved in the spinothalamic tract. Furthermore, diffusion tensor imaging data analysis was used to discover that fibers originated in the left thalamus to numerous regions of interest, adhered to brain anatomy, and coincided with the central thalamic radiation in migraine patients without aura (Li et al., 2015). Another study conducted a 5-week acupuncture treatment and suggested that increased N-acetyl aspartate/creatine in the bilateral thalamus post treatment, as well showed its correlation with the reduction in headache intensity (Gu et al., 2018). This study indicates that acupuncture may affect nociceptive modulation by altering neuronal activity in the thalamus and that alterations in the thalamus seem to be involved in acupuncture-related neural mechanism interpretations.

#### Insula

Studies have demonstrated abnormal insular networks, altered ALFF values between migraine patients and HCs (Zhao et al., 2014; Wei et al., 2022), and differentiated gray matter volume between chronic and episodic migraine (Valfrè et al., 2008). Focusing on the instant effects of acupuncture, Wei et al. suggested connectivity alterations in the right insular subregions during acupuncture treatment at GB8. Increased FC has been reported between the dorsal anterior insula and the right postcentral gyrus, together with decreased FC between the posterior insula and left precuneus, compared with those treated before (Wei et al., 2022). Focusing on the long-term effect of acupuncture, Zhao et al. (2014) reported an increased ReHo value in the bilateral insula only in the active acupoint group, which indicated an association between decreased VAS scores. In addition to the instant and sustained effects, placebo effects were also shown through the insula, which may be through relieving emotional disorders using sham acupuncture (Zhang et al., 2021). The insula plays a significant role in the emotionally related brain system and is associated with emotionally driven pain processing (Ostrowsky et al., 2002). Therefore, previous studies focused on instant and sustained acupuncturing effects on the insula, and further research may focus on their correlations with anxiety and depression symptoms in migraine headaches.

#### Amygdala

The amygdala is a core region of the neurolimbic system in which right lateralization plays a dominant role in the modulation of the top-down nociceptive pathway, pain processing, and negative emotion as well (Maizels et al., 2012; Minen et al., 2016). Recurrent nociceptive input during migraine was reported to affect the anatomical pattern of the amygdala, which may explain its altered function (Jia and Yu, 2017). Recently, fMRI studies have shown that disorders of amygdala-cortical interactions in migraine patients are likely to contribute to pain-related decision-making, cognitive impairment, and the process of pain chronicization. However, there is a lack of studies focusing on acupuncture altering the neuroplasticity of the amygdala. Only one study conducted by Tian et al. (2021) reported increased FC with the middle frontal gyrus, which correlated with a decreased VAS score after acupuncture treatment in MwoA patients. Moreover, the amygdala activated by negative emotions (Feinstein et al., 2011) may force the brain to focus on pain-related emotions, and studies have reported diminished negative FC (Etkin et al., 2010) and positive FC (Pezawas et al., 2005) between the amygdala and DMN in patients experiencing emotional stimuli (Kim et al., 2011). It is hoped that further studies should focus on the acupuncturing effects on amygdala activity and mechanisms focused on the rebuilding of DMN and limbic connections, which are likely to contribute to acupuncture in altering emotion-related impairment in migraine.

#### Right fronto-parietal network

The right fronto-parietal network strongly relates to the perception-somesthesis-pain pathway, one recent study reliably identified the changes within the rFPN in FCs by ICA in migraine (Smith et al., 2009; Li et al., 2017). The following studies have reported that impaired rFPN FCs could be reversed by longitudinal acupuncture (Li et al., 2015, 2017). Li et al. (2017) showed a significantly reduced FCs with the precuneus for rFPN after acupuncture in patients with MwoA, which was associated with greater headache intensity relief. In contrast, increased FCs were reported by 4-week standard acupuncture in another study, and the increased post-treatment FCs were positively correlated with VAS scores reduction (Li et al., 2015). Two studies showed a leading question is whether bilateral alterations of the connectivities within the rFPN have effects on acupuncture pain relief. The angular gyrus is a part of the inferior parietal lobe and is regarded as a perception-to-recognition-to-action interface based on its location and multiple connections (Seghier, 2013). Li et al. (2015) also showed activation of the angular gyrus post-treatment and its correlation with changes in migraine duration. Although the exact role of the angular gyrus in pain processing remains unclear, it indicates the function of the rFPN in top-down modulation and even the long-term acupuncturing effect in rFPN. Overall, the above studies underpinned new insights into the neural responses in rFPN induced by acupuncture and suggested its potential in modulating the perception-somesthesis-pain pathway in migraine treatment.

#### The occipital-temporal cortex

Visual, auditory, and olfactory auras are often preceded by migraine headaches. The occipital-temporal lobe is responsible for the multisensory integration of various visual, auditory, and tactile stimuli (Beauchamp, 2005; Amedi et al., 2007). Several previous studies have compared migraine patients with HCs, implying structural (Zhang et al., 2017; Chen et al., 2018) and functional abnormalities (Kreczmański et al., 2019; Tu et al., 2019) of the middle occipital cortex, and reported correlations between the neuroimaging alterations and clinical index (Zhao et al., 2013). Furthermore, a series of recent machine learning studies illustrated that abnormal activity in the bilateral middle occipital gyrus could not only be used as a diagnostic marker to classify MwoA patients (Tu et al., 2020), but can also predict the response to acupuncture treatment as well (Yin et al., 2020). A further study focused on the acupuncturing effects showed increased FC between the superior temporal gyrus and the precuneus after verum acupuncture (Zhang H. et al., 2016; Li et al., 2017). Similar results were reported by Yin et al. (2020), who suggested that after 20 sessions of acupuncture treatment, the migraine group showed diminished ALFF values in the right and left occipital gyrus, and positively correlated with the relief of VAS scores and the reduction of mean migraine days. Migraines have been shown to be associated with deficient visual evoked potentials or increased intensity dependence of auditory evoked potentials (Ambrosini et al., 2017), which may directly demonstrate the altered activation of the dysfunctional occipital-temporal cortex. Since imbalance in the occipital-temporal cortex may be related to deficits in multi-sensory processing during interictal periods, we are looking forward to the acupuncturing effects being further discussed in terms of altering the neuroplasticity of the occipital-temporal cortex or the evoked VEP/IDAP.

#### Cerebellum

Studies have revealed that in addition to motor task involvement, the cerebellum plays a significant role in pain processing and regulation (Moulton et al., 2010, 2011). A study on patients with cerebellar infarction showed activated perception of heat and repeated mechanical stimuli, and an absence of active endogenous pain inhibitory mechanisms suggested an undervalued role of the cerebellum in hypalgesia (Ruscheweyh et al., 2014). Besides, clinical studies showed that after acupuncture treatment sessions, there were consistently increased ReHo values in the cerebellum and other brain regions and there were correlated with a decreased VAS score (Zhao et al., 2014; Liu et al., 2021b). Other studies have reported increased FCs with the precuneus and decreased metabolism in the cerebellum after sustained and instant acupuncture (Yang et al., 2012; Li et al., 2017). In accord with these findings, a clinical trial based on machine learning showed that the Fisher’s z transformation ALFF value of the foci in the left superior cerebellum could distinguish patients with MwoA from HCs with an accuracy of up to 80% (Yin et al., 2020). Abnormal cerebellar activation was reported in various subtypes of migraine patients, and further research has indicated its abnormally decreased control function in the DPMS and thalamic sensory gating. However, further studies are required to focus on the cerebellum in the mechanism of acupuncture analgesia.

## Discussion

Clinical trials have demonstrated that acupuncture may be beneficial for the treatment of migraines. Benefits are mainly embodied in the reduction of the headache intensity (VAS, SF-MPQ, and Six-Point Likert Scale), migraine attack duration, headache frequency, the number of days with headache, the days of acute-medication intake, the reduction of comorbidities and disability, and the enhancement of the quality of life. However, the exact biochemical and neural mechanisms underlying acupuncture analgesia remain unclear, although there is little evidence of acupuncture preventing neuroinflammation and relieving neuronal sensitization.

As presented in Figure 3, we inferred from basic experiments that acupuncture may play protective effectiveness on neurons through several ways: (1) acupuncture may have the capacity to moderate neuroinflammation by reducing the release of trigeminal-activated neuropeptides (GCRP, SP, and PACAP), inhibiting dural immune cells (macrophages and mast cells), downmodulating inflammatory related mediator levels (PGE2, IL-1β, COX2, IL-6, TNF-α, VIP, ET, and MLCK); (2) acupuncture may have the capacity to reduces neuronal sensitization by reducing cytokine level (BDNF, Glutamate), relieving neuronal activation in migraine-related brain areas, and modulating endocannabinoid and serotonin system.

**FIGURE 4:**
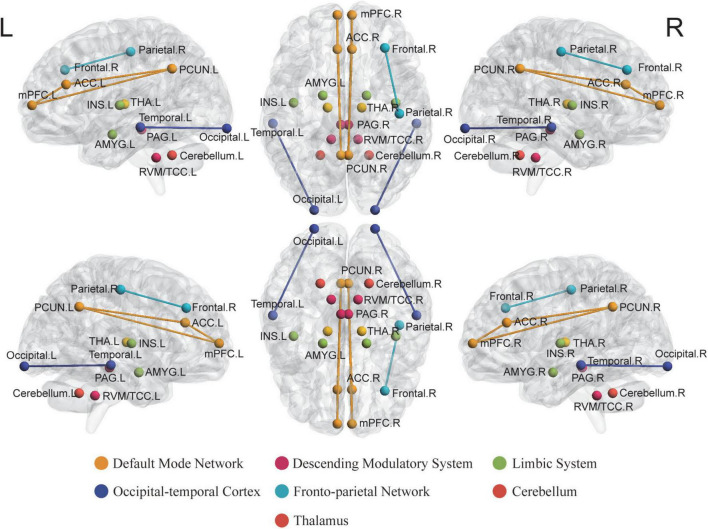
The main findings of acupuncture for migraine by neuroimaging techniques. The high frequent reported areas that have been affected by acupuncture for migraine in the included studies were noted with balls in different colors. Different colors of the nodes and edges represent different brain regions and networks. ACC, anterior cingulate cortex; AMYG, amygdala; INS, insula; MFG, middle frontal gyrus; MTG, medial temporal gyrus; PAG, periaqueductal gray; PCUN, precuneus; mPFC, medial prefrontal cortex; RVM, rostral ventromedial medulla; TCC, trigeminal cervical complex; THA, thalamus.

Acupuncture has been discussed to have nonspecific modulation effects on a number of brain regions, implicated in the regulation of nociceptive perception and emotional disorders (e.g., anxiety and depression). And it may have its exclusive neural mechanism in relieving pain, comorbidities, and cutting down aura occurring, thus elevating the life quality of patients suffering from migraine. We summarized the brain alteration after acupuncture reported by articles and illustrated the regions and networks. Altered Regions were involved in pain perception (e.g., the DPMS, thalamus, limbic system, rFPN, and cerebellum), nociceptive emotional processing (e.g., the DMN and amygdala), and aura occurring (e.g., the occipital-temporal cortex) of migraine (Figure 4). However, a more distinct combination of brain regions or a more specific way of alteration is expected in further neuroimaging research, which may deepen our discussion over acupuncturing effects.

## Limitations and perspectives

The present study has several limitations, including the limited number of studies, absence of sham acupuncture controls, and risks of bias, which may all affect the results of our review. However, further rigorous acupuncture clinical trials should consider the following improved methodology: (1) Due to lack of instructions during scanning or low statistical power (small sample sizes), studies resulted in high probability of false positives in the fMRI data (Grady et al., 2021). Replicated and multi-center brain imaging studies are required to identify neuroplasticity alterations and acupuncture effects, indicating further possible acupuncture mechanisms. (2) Cross-validation of the derived neuroimaging results requires further multi-modality brain imaging studies comprising diverse data analysis methodologies. (3) More studies may further detect the functional changes at specific points and independent meridians’ effects and their correlation with migraine reduction. (4) Machine learning methods in neuroimaging research may assist in predicting acupuncture effects and evaluating acupuncture responders.

## Author contributions

LL conceived and designed the review. YC, YL, and LL drafted the manuscript, designed the tables, and designed the figures. LL, YS, and SZ modified the language and checked the text. JS and BL checked the figures and tables and confirmed the information. All authors read and approved the final manuscript.

## Abbreviations

5-HT: 5-hydroxytryptamine
5-HT7R: 5-hydroxytryptamine7 receptor
ACC: anterior cingulate cortex
ALFF: amplitude of low frequency
BDNF: brain-derived neurotrophic factor
CB1: cannabinoid 1
CGRP: calcitonin gene-related peptide
CM: chronic migraine
CNS: central nervous system
COX2: cyclooxygenase-2
DMN: default mode network
DPMS: descending pain modulatory system
DTI: diffusion tensor imaging
ET: endothelin
EX-HN: Tojingbu Xue Points of Head and Neck
FCs: functional connectivities
fMRI: functional magnetic resonance imaging
GB: gallbladder meridian
GV: governor vessel
HCs: healthy controls
ICA: independent component analysis
IL-1 β: interleukin-1 β
IL-6: interleukin-6
LI: large intestine meridian
LR: liver meridian
MA: manual acupuncture
MLCK: myosin light chain kinase
mPFC: medial prefrontal cortex
MSQ: Migraine-Specific Quality of Life Questionnaire
MwoA: migraine without aura
NO: nitric oxide
PACAP: pituitary adenylate cyclase-activating polypeptide
PAG: periaqueductal gray
PGE2: prostaglandin E2
PPE: plasma protein extravasation
rACC: rostral anterior cingulate cortex
mPFC: medial prefrontal cortex
RCTs: randomized clinical trials
ReHo: regional homogeneity
rFPN: right fronto-parietal network
rsfMRI: resting-state functional magnetic resonance imaging
RVM: rostroventromedial medulla
SBA: seed-based analysis
SF-36: Short Form-36 Health Survey Scale
SF-MPQ: McGill Pain Questionnaire Short Form
SP: spleen meridian
SP: substance P
ST: stomach meridian
TCC: trigeminocervical complex
TG: trigeminal ganglion
TNC: trigeminal nucleus caudalis
TNFα: tumor necrosis factor-α
VAS: visual analog scale
VIP: vasoactive intestinal peptide
vlPAG: ventrolateral periaqueductal gray
VN: vagus nerve
WDR: wide dynamic range.
