# Supplementary material for: Therapeutic applications and potential mechanisms of acupuncture in migraine: A literature review and perspectives
Source: Front Neurosci. 2022 Oct 20;16:1022455. doi: 10.3389/fnins.2022.1022455 (PMC9630645; doi:10.3389/fnins.2022.1022455)
Supplement: Supplementary file 1 [file Table_1.docx]

Table 1: The characteristic of current clinical trials of acupuncture in the treatment of migraine

| Study | Clinical trial design | Clinical condition | Intervention | Comparison | Acupoints | Acupuncture parameters | Outcomes | Sham acupuncture technique |
| --- | --- | --- | --- | --- | --- | --- | --- | --- |
| Xu et al. (2020) | RCT | Episodic  migraine | Acupuncture (n=60) | Sham acupuncture (n=60)  Usual care (n=30) | Bilateral L14, LR3, EX-HN5, GB20, and GB8  (addition: bilateral ST8, BL10, GV20) | Deqi, 30 minutes, manipulate 10 seconds for 4 times, once | **Primary outcomes:**  Migraine days and attacks (week13-20) ↓  **Secondary outcomes:**  Higher responder rates, VAS↓  MSQ↑ | Non-penetrating sham acupuncture at heterosegmental non-acupuncture points plus usual care |
| Giannini et al.  (2020) | Comparison trail | Episodic migraine | Acupuncture (n=69) | Pharmacological (n=66) | Bilateral LR3, GB34, SP6, LI4, TE5, GV20  (addition: ST8, BL2, GB4, GB 8, GB20, BL12) | 12 sessions, 2 sessions for first week, weekly for next 10 weeks | NS |  |
| Nie et al.  (2019) | RCT | Migraine | Acupuncture& tuina(n=45) | Acupuncture (n=45)  flunarizine hydrochloride (n=45) | Bilateral ST8, EX-HN5, GB8, GB20, GB11; EX-HN3, GV20  (additional: GV23, CV12; bilateral IL4, ST36, SP6, ST40, SP9, LR3, KI3, GB43, PC6, SP10, BL17) | 10-15 mm, deqi, 30 minutes, twice a week in the first 4 weeks, once a week during weeks 5–8, once every 14 days during weeks 9–12 | NS |  |
| Yu et al.  (2018) | RCT | MM | Acupuncture (n=7) | Acupressure (n=6)  Control acupuncture (n=5) | Bilateral LR3, LI4, SP6, GB20  (additional: bilateral: ST36, SP10, KI3, LR2) | 15-20mm, deqi, 20 minutes, 1-2 full rotations per 10 minutes at 4 times per second,  3 months, 3 times per month | Mean migraine days； average pain; peak pain (4th cycle-month than 2nd and 3rd ); total duration period of MM (4th cycle-month than 2nd)↓  Patients with≥50% reduction |  |
| Tastan et al.  (2018) | Comparison trail | Migraine | Acupuncture (n=30) | Hypnotherapy (n=30)  Pharmacotherapy (acetaminophen 650 or 1300 mg)(n=30) | Bilateral LI4, LU7, SI3, ST6, ST8, ST36, SP6, KI3, TE5, TE18, BL2, BL12, BL60, GB20, LR2, LR3; EX-HN5, GV14, GV20, EX-HN3  ear ShenMen, ear hypothalamus, and ear antidepressant points | 0.5 cun, 30 minutes, 3 days a week, 10 sessions | VAS, MIDAS↓ |  |
| Musil et al.  (2018) | RCT | Migraine | Acupuncture (n=42) | Waiting-list control group (n=44) | Bilateral: GB20, EX-HN5, GB8, LI4  Optional points:  Bilateral LR2, LR3, KI3, GB39, SP6, ST40, SP9, SP6, SP10; GV20, GV23, CV20, Ashi point | 10-30 mm, deqi, 25minutes, 14sessions, 12 weeks | **Primary outcomes:**  Migraine days↓  **Secondary outcomes:**  Responders↑;  symptomatic medication intake, MIDAS↓  number of patients with mild vs moderate and severe before and after the intervention↓ |  |
| Farahmand et al.  (2018) | RCT | Acute migraine | Auricular acupuncture (n=30) | Sham acupuncture (n=30) | Ear acupoints: bilateral shenmen, autonomic, thalamus, frontal, temple | 10 minutes, once  (nonrespondent-given rescue medicine) | VAS score under 15, 30, 45, and 60 minutes↓ | Similar needles inserted into unusual points |
| Zhao et al.  (2017) | RCT | Migraine | Acupuncture (n=83) | Sham acupuncture (n=80)  Waiting list group (n=82) | GB20, GB8  (additional: TE5, GB34, BL60, SI3, LI4, ST44, LR3, GB40)  Alternatively unilateral | Deqi, nerve stimulator, 4 weeks, 5 sessions per week  EA: 2/100 Hz, altering every 3 seconds, 0.1-1.0 mA | **Primary outcomes:**  frequency of migraine attacks↓  **Secondary outcomes:**  migraine days, VAS↓ | No deqi sensation; Four nonpoints |
| Naderinabi et al.  (2017) | RCT | CM | Acupuncture (n=50) | Botulinum toxin A injection (trigger zones) (n=50)  Controls (sodium valproate)(n=50) | Bilateral；GB41, GB 20, GB15, GB14, GB10, GB8, LI4, LR3, TE5, GV20;  Additional: bilateral EX-HN5 | 10-15 mm, deqi, 30 sessions in 60 days | VAS; days with migraine; acute medication; rate of side effect↓ |  |
| Gaff et al.  (2016) | Comparison trail | Pediatric migraine | Auricular acupuncture(n=19) | Self-controlled | Efficacious points on 2 proposed auricular migraine lines | 15 minutes, a maximum of 3 needles (ASP gold needle) in each ear, once | VAS↓ |  |
| Zheng et al.  (2015) | RCT | Migraine | Acupuncture (n=558)  Shaoyang-specific (n=121)  Shaoyang-non-specific (n=119)  Yangming-specific (n=118) | Sham acupuncture (n=118) | Unilateral  Shaoyang-specific: TE5, GB34, B40, GB20;  Shaoyang-non-specific:  TE19, TE8, GB33, GB42;  Yangming-specific: ST8, LI6, ST36, ST42; | deqi, 30 minutes, 4 weeks, 5 sessions a week, | **Correlation:**  participants with higher post-treatment expectation levels (50%, 75% and 100%), but not pretreatment expectation levels, had significantly fewer NDMA days and lower VAS scores than those with lower expectations (0% and 25%). | Penetrating needles at non-acupuncture points |
| Wang et al.  (2015) | RCT | Frequent migraine | Acupuncture (n=26) | Sham acupuncture (n=24) | Bilateral: GB20; Unilateral: EX-HN5, GB8, LI4,  Additional bilateral: GV20, LR2, LR3, KI3, GB39, SP6; GV20, GV23, ST36, SP6; ST40, CV12, SP9; SP6, SP10, Ashi point | 10-30mm, Deqi, 25 minutes, stimulation every 10 minutes, two sessions per week (0-4w), once per week (5-8w), once every two weeks (9-12w), once per month (13-20w) | **Primary outcomes:**  Migraine days, Six-Point Likert Scale↓,  Responders (after and 3M follow-up), faster reduction of migraine pain  **Secondary outcomes:**  MSQOL (FR, EF)↑;  MSQOL (FR, FP, EF), MQS↓(3M follow-up)  faster improvement on FP and EF  Number of participants who took pain killers (VA<SA)  significantly higher PPTs in the 11 sites except for bilateral EX-HN5 |  |
| Hou et al.  (2015) | RCT | Migraine (episodic and chronic) | BoNTA injection (n=42) | Fixed (muscle)-sites BoNTA injection (n=41)  Placebo injection (n=19) | EX-HN3, GV20; bilateral EX-HN5,  GB8, GB20, BL10 | 0.1 mL of saline containing BoNTA 2.5 U, 1-inch, 30-gauge needle, once | Attack frequency of migraine, VAS, mean duration of each attack, migraine-associated symptoms (1-4 month)↓ |  |
| Rezvani et al.  (2014) | Comparison trail | Migraine | Classic acupuncture (n=40) | Yamamoto new scalp acupuncture (n=40) | Bilateral: LI4, ST8, ST36, BL2, GB14, TB5, GB8, EX-HN5, SI3, BL10, BL60, GB20, LIV3, KID1, GV20, sishencong, LU7, SP6, LIV2, ST36, REN6 | 10-15 mm, deqi, 30 minutes, 18 sessions, every other day | NS |  |
| Foroughiour et al.  (2014) | RCT | Migraine | Acupuncture (n=50) | Sham acupuncture (n=50) | According to involved meridians and Chinese medicine syndrome differentiation | Deqi, 30 minutes, 4 weeks, 3 sessions per week | Headache attack frequency↓ | Needled superficially |
| Cayir et al.  (2014) | Controlled trail | Migraine | Acupuncture (n=27) | Self-controlled (n=27) | Bilateral ST8, ST44, LI4, LI11, LIV3, SP6, GB1, GB14, GB20, GV14, GV20, EX-HN5, ear Shenmen; EX-HN3 | 0.5-1 cm, 30 minutes, no seek for deqi, 5 weeks, 2 sessions per week | VAS, MMP-2 activity (1st and last session)↓  SF-36↑ |  |
| Yang et al.  (2013) | RCT | CM | Acupuncture (n=33) | Topiramate (n=33) | Bilateral BL-2, GB-20, EX-HN5; EX-HN3 | Deqi, 30 minutes, 12 weeks, 2 sessions per week | Patients with throbbing symptoms had better prognosis with acupuncture than those without throbbing; |  |
| Wang et al.  (2012) | RCT | Acute migraine | Acupuncture (n=75) | sham acupuncture (n=75) | Bilateral ST8, GB8, GB20; GV20, GV24  Additional: bilateral  Shaoyang headache: TE5, GB34; Yangming headache: LI4, ST44; taiyang headache: BL60, SI3; Jueyin headache: LR3, GB40; nausea and vomiting: PC6; dysphoria and susceptibility to rage: LR3 | 10-15 mm, deqi, 30 minutes, once | VAS, SF-MPQ, acute medication intake (48h), accompanying symptoms (24, 48, 72h)↓ | 1) acupoints unrelated to headache  2) were located 3 mm apart from the selected acupoints;  3) these 30 sham points were randomly assigned to five subgroups of sham acupuncture group , and recorded in the predetermined computer-made randomization sealed envelope;  4) Sham points were all punctured perpendicularly. |
| Wallasch et al.  (2012) | RCT | Migraine | Acupuncture (n=18) | Sham acupuncture (n=17) | Bilateral: LI4, ST36, TE5, GB41, SI3, UB62, GB20, EX-NH5, TE23, LI3, KI3; GV20 | 30 minutes, 8 weeks, once a week | Days with migraine, duration of migraine (h/month)↓ | Outside a classically described acupuncture point; inserted superficially |
| Li et al.  (2012) | RCT | Migraine | Acupuncture (n=360)  Shaoyang-specific group (n=121)  Shaoyang-nonspecific group (n=119)  Yangming-specific group (n=118) | Sham acupuncture (n=118) | Bilateral  Shaoyang-specific: TE5, GB34, GB40, GB20;  Shaoyang-nonspecific: TE19, TE8, GB33, GB42;  Yangming-specific: ST8, LI6, ST36, ST42 | EA: 2 Hz, 100 Hz, 0.1-1 mA, Deqi, 30 minutes, 4 weeks, 5 sessions per week | Shaoyang-specific vs. sham:  MSQ↑, migraine duration (5-8w, 13-16w), migraine frequency (1-4w, 5-8w, 13-16w), VAS (1-4w, 5-8w, 13-16w), intensity of migraine (5-8w, 13-16w)↓  Shaoyang-nonspecific vs. sham:  migraine duration (1-4w, 13-16w), migraine frequency (1-4w, 5-8w, 13-16w), VAS (13-16w)↓  MSQ-restrictive (1-4w, 13-16w), MSQ-preventive↑  Yangming-specific vs. sham:  migraine duration (13-16w), migraine frequency (1-4w, 13-16w), VAS (13-16w)↓ | No deqi sensation |
| Ferro et al.  (2012) | RCT | Women with CM | Acupuncture (n=16) | Tanacetum (n=16)  Acupuncture+ tanacetum (n=16) | Bilateral GB8, GB14, HT7, LR2, LI4; GV20 | Deqi, 30 minutes, 10 weeks, 2 sessions per week | SF-36↑  MIDAS, VAS↓ |  |
| Yang et al.  (2011) | Comparison trial | CM | Acupuncture (n=33) | Topiramate (n=33) | Bilateral BL-2, GB-20, EX-HN5; EX-HN3 | Deqi, 30 minutes, 12 weeks, 2 sessions per week | **Primary outcomes:**  headache days, moderate/severe headache days, MIDAS, BDI-Ⅱ, HADS, days of acute-medication intake↓  SF-36↑  **Secondary outcomes:**  ≥50% decrease in the number of headache and moderate/severe headache days |  |
| Wang et al.  (2011) | RCT | MwoA | Acupuncture+ placebo (n=70) | Sham acupuncture+ flunarizine (n=70) | GV20, GV24; bilateral GB13, GB8, GB20  Additional:  TE5, GB34 (TE-GB);  LI 4, ST 44;  BL60, SI 3;  LR3, GB40;  PC6;  LR3 | 10-15 mm, deqi (5 to 10 times of lifting, thrusting, and twirling), 30 minutes, 4 weeks, 3 sessions per week | **Primary outcomes:**  responder rates↑  **Secondary outcomes:**  Number of migraine days, acute medication↓ | 1) acupoints unrelated to headache  2) were located 3 mm apart from the selected acupoints;  3) these 30 sham points were randomly assigned to five subgroups of sham acupuncture group , and recorded in the predetermined computer-made randomization sealed envelope.  4) Sham points were all punctured perpendicularly. |
| Allais et al.  (2011) | RCT | MwoA | Auricular acupuncture (positive area) (n=46) | Auricular acupuncture (unsuitable area) (n=48) | The tender points | If the pilot test for tender points was positive and the reduction was at least 25% in respect to basis, a semi  permanent needle (ASP SEDATELEC, France) was  inserted after 1 min. | VAS (10, 3, 60 ,120 min after needle insertion)↓ |  |
| Li et al.  (2009) | RCT | Migraine | Acupuncture (n=58) | Sham acupuncture 1 (n=60)  Sham acupuncture 2 (n=57) | Bilateral TE5, GB 34, GB 40, TE 20, GB 20 | Deqi, 30 minutes, once | **Primary outcomes:**  VAS↓(2,4 h)  **Secondary outcomes:**  Rate of pain relief, general effectiveness evaluation;  relapse or aggravation within 24 hours↓ | Needling at predesignated nonacupoints |
| Jia et al.  (2009) | RCT | Migraine | EA (n=138) | Control group (n=137) | Bilateral GB40 | 15-20 mm, obvious needling sensation, 100 Hz, 30 minutes, once a day for 4 courses, 2-days interval in between | VAS↓  better effects in 4-week and 6-month follow-up (improve in integral scale)  5-HT level↑ |  |
| Facco et al.  (2008) | RCT | MwoA | Acupuncture (n=32) | Ritualized mock acupuncture+ rizatriptan (n=30)  standard mock acupuncture+ rizatriptan (n=31)  rizatriptan (n=34) | Each type of syndrome owns specific acupoint selection according to TCM | 30 minutes, deqi, large rotation at a rate of 3 rotations/second, 20 sessions, 2 session per week (1-week rest after 10 sessions), | Rizatriptan intake (from 3 to 6 M)↓ |  |
| Bäcker et al.  (2008) | RCT | Migraine | Acupuncture (n=17) | Sham acupuncture (n=13) | Bilateral GV20, GB20, EX-HN5, TE23, TE5, LR3, GB41 | TE 5, GB 41, LR 3: during 2 minutes of paced breathing;  Gb 20, Taiyang, Gv 20: needled directly after the paced breathing episode;  GB 41, LR 3, TE 5, GB20: manually rotated with a frequency of 2 to 4 Hz, amplitude of 90 to 120 degrees, deqi, 30 minutes | High-frequency power of heart-rate variability↓ | Outside a classically described acupuncture point were chosen; inserted superficially |
| Alecrim-Andrade et al.  (2008) | RCT | Migraine | Acupuncture (n=19) | Sham acupuncture (n=17) | Differs from session to session | Deqi,30 minutes, 16 sessions | **Primary outcomes:**  percentage of patients with a ≥50% reduction;  **secondary outcomes:**  number of days with migraine per month (2nd M);  percentage of patients  with ≥40% reduction in migraine attack frequency (1st and 2nd M) | Superficial insertion in acupuncture points with needles almost falling out; no manipulation was done; The points were selected after an extensive consultation of the Chinese acupuncture literature with no references to effects on headaches |
| Streng et al.  (2006) | Comparison trail | Migraine | Acupuncture  (n=59) | Metoprolol (n=55) | Bilateral GB20, GB40 or GB41 or GB42, GV20, LI3, TE3 or TE5, EX-HN5 | Deqi, 20-30 minutes, 12 weeks, 8-15 sessions | **Secondary outcomes:**  Week 12:  SES, PDI, average pain scale (0-10 score)↓  SF-36↑  Week 24:  Number of migraine attacks, ≥50% reduction migraine attacks, SES, PDI, average pain scale↓  SF-36 (physical health)↑  fewer adverse effects |  |
| Diener et al.  (2006) | RCT | Migraine | Acupuncture  (n=313) | Sham acupuncture (n=339)  Standard therapy (n=308) | Based on traditional Chinese medicine diagnosis for syndromes , acupuncture channels related to the individual headache area, and Ah Shi points | 2-10 mm, deqi, 30 minutes, 10 sessions in 6 weeks | NS |  |
| Alecrim-Andrad et al.  (2006) | RCT | Migraine | Acupuncture (n=14) | Sham acupuncture (n=14) | BI10; bilateral GB12, GB20, GB21;  Occipital headache: BL60, SI3;  Frontal headache: BL2, ST36, GV23, LI4;  Temporo-pariental headache and orbitary headache and hemicranias: TE5, GB34, GB8;  Holocranea/uphead: SI3, GV20, LV3;  Anxious patients: P6  Patients with liver symptoms: LV3 | Deqi, 16 sessions in 12 weeks, twice a week during the first 4 weeks  and weekly during the following 8 weeks | NS | Minimal acupuncture was used and consisted of very shallow needle insertion in the acupuncture points.  2No manipulation  3 confirm minimal or no influence on headaches |
| Linde et al.,  (2005) | RCT | Menstrually related migraine | Acupuncture (n=15) | sham acupuncture (n=13) | GB8, GB20, LI4, LR3, SP6;  Additional  GB14, EX-HN5,UB10, | 10-30 mm, deqi, 30 minutes, 3 month | NS | Blunt placebo needles that touch but do not penetrate the skin |
| Linde et al.  (2005) | RCT | Migraine | Acupuncture (n=145) | Sham acupuncture (n=81)  Waiting list (n=76) | Bilateral GB20, GB40 or GB41 or GB42, GV20, LI3, TE3 or TE5, EX-HN5  Additional: according to symptoms | 30 minutes, 12 sessions in 8 weeks | NS | Distant nonacupuncture points |
| Melchart et al.  (2003) | RCT | Acute migraine | Acupuncture (n=60) | Placebo (1 mL NaCl solution)(n=61)  Sumatriptan (n=58) | GB20, GB15, GB41 or GB14, GB10, GB8, GV20, LI3, LR3 or TE5, EX-HN5 | Deqi, 1.5h, once | Sumatriptan was more effective than acupuncture at relieving headache in the first 2 h |  |
| Allais et al.  (2003) | Comparison trail | CM | Acupuncture (n=20) | TENS (n=20)  Infrared lasertherapy (n=20) | LR3, SP6, LI4, GB20, GV20, EX-HN5  Additional:  Based on symptoms | twice a week for the first 4 weeks and once a week for the next 6 weeks | Number of headache days↓(3rd and 4th M) |  |
| Allais et al.  (2002) | Comparison trail | MwoA | Acupuncture (n=80) | Flunarizine (n=80) | Bilateral LR3, SP6, ST36, CV12, LI4, PC6, GB20; GB14, EX-HN5, GV20 | 10-30 mm, deqi, weekly sessions for the first 2 months and then once a month for the next 4 months | Number of attacks (2nd and 4th M), analgesic consumption (2nd M), Pain intensity↓  less side effect |  |
| Pintov et al.  (1997) | RCT | Children with migraine | Acupuncture (n=12) | Sham acupuncture (n=10)  HC (n=10) | - | Needle inserted in the dermis, once | Panopioid activity, β-endorphin level in plasma↑ | Inserted in stratum corueum |
| Hesse et al.  (1994) | RCT | Migraine | Acupuncture with placebo tablets (n=38) | Placebo stimulation with metoprolol (n=39) | Determined individually | 1-3 weeks between treatments  6-8 treatments in 17 weeks. | Global rating of attacks↓ |  |
| Vincent et al.  (1989) | RCT | CM | Acupuncture (n=15) | Sham acupuncture (n=15) | LR3, EX-HN5,GB20 or BI10, | 1-2 cm, 15 minutes, 10s of stimulation every 5 minutes | Pain score (six-point pain scale), medication intake↓ | Depth of insertion was 2 mm,which is just sufficient to make the needle stand vertically; stimulation was very light |

CM: chronic migraine; MM: menstrual migraine; MwoA: migraine without aura; HC: healthy controls; VA: verum acupuncture; SA: sham acupuncture; NS: no significant; VAS: visual analogue scale; MSQ: migraine-specific quality of life questionnaire; MIDAS：Migraine Disability Assessment scale; PPTs: pressure pain threshold; BoNTA: Onabotulinumtoxin A; SF-36: Short Form-36; HADS: Hospital Anxiety and Depression Scale; SES: Self-Esteem Scale; PDI: Pain Disability Index; 5-HT: 5-hydroxytryptamine; SF-MPQ: McGill Pain Questionnaire Short Form; BDI: Beck Depression Inventory; rp: responders; nrp: non-responders; NS: no significant; GB: gallbladder meridian; ST: stomach meridian; LI: large intestine meridian; GV: governor vessel; EX-HN: Tojingbu Xue Points of Head and Neck; LR: liver meridian; SP: spleen meridian; PC: pericardium meridia; TE: triple energizer meridian; BL: bladder meridian; CV: conception vessel; KI: kidney meridian.
